# Synthetic complex Weyl superconductors, chiral Josephson effect and synthetic half-vortices

**DOI:** 10.1038/s41598-023-44910-0

**Published:** 2023-10-20

**Authors:** Zahra Faraei, Seyed Akbar Jafari

**Affiliations:** 1 Department of Physics, Institute for Advanced Studies in Basic Sciences (IASBS), Zanjan, 45137-66731 Iran; 2 Sharif University of Technology, Department of Physics, Tehran, 11155-9161 Iran; 3 Institute for Advanced Simulations, Forschungszentrum Jülich, 52425 Jülich, Germany

**Keywords:** Physics, Condensed-matter physics, Superconducting properties and materials, Topological matter

## Abstract

We show that the most generic form of spin-singlet superconducting order parameter for chiral fermions in systems with broken time reversal symmetry and inversion symmetry is of the $\Delta _s+i\gamma ^5\Delta _5$ where $\Delta _s$ is the usual order parameter and $\Delta _5$ is the pseudo-scalar order parameter. After factoring out the *U*(1) phase $e^{i\phi }$, this form of superconductivity admits yet additional complex structure in the plane of $(\Delta _s,\Delta _5)$. The polar angle $\chi$ in this plane, which we call the chiral angle, can be controlled by the external flux bias. We present a synthetic setup based on stacking of topological insulators (TIs) and superconductors (SCs). Alternatively flux biasing the superconductors with a fluxes $\pm \Phi$ leads to $\Delta _5=\Delta _0 \sin (\chi )$, where $\Delta _0$ is the superconducting order parameter of the SC layers, and the chiral angle $\chi ={2\pi }\Phi /\Phi _0$ is directly given by the flux $\Phi$ in units of the flux quantum $\Phi _0=h/(2e)$. This can be used as a building block to construct a two-dimensional Josephson array. In this setup $\chi$ will be a background field defining a pseudoscalar $\Delta _5$ that can be tuned to desired configuration. While in a uniform background field $\Delta _5$ the dynamics of $\phi$ is given by standard XY model and its associated vortices, a *staggered* background $\pm \Delta _5$ (or equivalently $\chi$ and $\chi +\pi$ in alternating lattice sites) creates a new set of minima for the $\phi$ field that will support half-vortex excitations. An isolated single engineered “half-vortex” in the $\chi$ field in an otherwise uniform background will bind a $\phi$-half-vortex. This is similar to the way a p-wave superconducting vortex core binds a Majorana fermion.

## Introduction

Weyl semimetals (WSMs) are a novel class of topological materials that host *chiral* fermions as their low-energy excitations^1–3^. The chirality is an additional attribute of the electrons in WSMs that defines whether they are eigenstates of the $\gamma ^5$ matrix with eigenvalues $+1$ and $-1$^3,4^. Having this extra attribute, the electrons in the WSMs will be described by four components. Extension of the phenomenological Ginzburg–Landau theory to a relativistic formalism^5–8^ reveals different forms of superconductivity in these materials^9–12^. Strictly speaking, the quartic fermionic expressions which represent the superconducting interactions can be made from scalar, pseudoscalar, vector, axial vector and tensor bilinear structures under Lorentz transformations (for more details refer to supplementary material). Practically, placing a three dimensional Dirac/Weyl semimetal in proximity to a conventional superconductor (with spin-singlet s-wave order parameter) can lead to all of these types of superconductivity due to the superconducting potential which is penetrated into the Dirac/Weyl matter^12–14^.

In this paper, we consider a model for Weyl superconductors that in addition to the conventional pairing (denoted by $\Delta _s$, with “s” subscript for scalar), simultaneously supports a novel form of pseudoscalar superconductivity denoted by $\Delta _5$ for obvious reasons. This model further allows one to adjust the values of the above two forms of order parameter by external flux bias^9^^9^. We follow the model of Meng and Balents^9^, who showed that a periodic stacking of magnetically doped topological insulator (TI) and a conventional superconductor gives rise to a Weyl superconductor. They also proposed the idea of flux biasing the superconductors with phases $\pm \chi$ to split each Weyl node in the spectrum into a pair of Bogoliubov Weyl nodes^15^. We show that tuning the phase $\chi$ enables us to realize not only the pure scalar^9^ and pure pseudoscalar^16^ superconductivity, but also a more interesting combination of them. The interplay of these two kinds of superconducting orders which are respectively even and odd under parity, leads to synthesis of half-vortices in a Josephson array composed of such superconductors. We will show that the phase variable $\chi$ (controlled by external bias) is given by the ratio of the pseudoscalar to the scalar component of the general superconducting order parameter and is the polar angle in the complex plane of $(\Delta _s,\Delta _5)$. This *chiral angle* (CA) plays a significant role in the Josephson energy and the Josephson current. The spatial variations of $\chi$ in a single Josephson junction lead to a chiral Josephson current which in some circumstances can be separated from ordinary non-chiral one.

This paper is structured as follows: First we introduce the synthetic setup for a tunable realization of a combination of scalar and pseudoscalar superconductivity. It is followed by a discussion of the properties of such a combination of scalar and pseudoscalar superconductivity. Then we study a single Josephson junction of this type of superconductors and show the non trivial dependence of Josephson energy to the CA difference of the two superconductors. In the next section we discuss the construction of half-vortices in an array of this type of Weyl superconductors and then we drive the chiral Josephson current in such arrays. The last section is devoted to summary and discussion.

**Figure 1:**
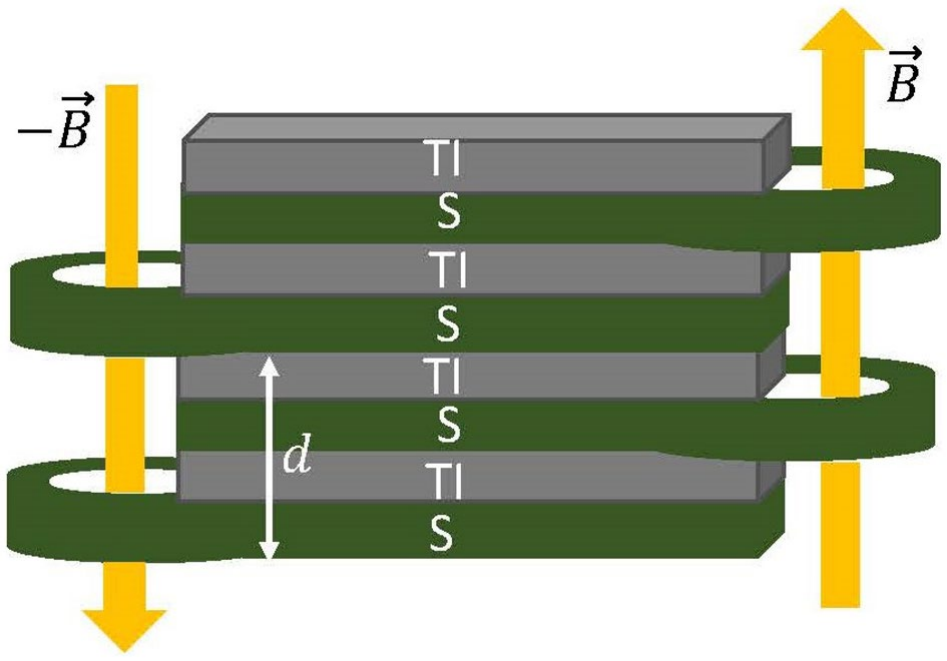
A schematic model of a synthetic $(\Delta _5,\Delta _s)$ superconductor realized by stacking superconductor (SC) and topological insulator (TI) layers with a periodicity of *d*. A magnetic field $\vec {B}$ induces a flux $\Phi$ through the superconductors, which modifies the phases of adjacent SC layers by $\phi \pm {2\pi } \Phi /\Phi _0$, where $\phi$ is the initial uniform phase of the superconductors and $\Phi _0$ is the flux quantum.

## Results

### A model for synthetic realization of $(\Delta _s,\Delta _5)$ superconductor

As Meng and Balents proposed in Ref. ^9^, alternating stack of (s-wave) superconductors(SCs) and topological insulators (TIs) realizes a Weyl superconductor. In this periodic structure, the tunneling parameter between the upper and lower layers of each TI ($t_S$) differs from the tunneling parameter of a TI layer to the next TI layer ($t_D$). This consideration in addition to a magnetization (*m*) in the direction perpendicular to the surface of the layers, lead to the formation of Weyl nodes at ${\vec k}=(0,0,\pi /d\pm k_0)$ where $k_0=1/d~\arccos (1-\frac{m^2-(t_S - t_D)^2}{2~t_S~t_D})$ and *d* is the period of the structure. The role of superconductors is to proximitize the coupling potential and split each Weyl node to two Bogoliubov Weyl nodes. Competition between $\Delta _0$ and *m* results in four Weyl nodes at ${\vec k}_=(0,0,\pi /d\pm k'_0)$ where $k'_0=1/d~\arccos (1-\frac{(m\pm \Delta _0)^2-(t_S - t_D)^2}{2~t_S~t_D})$. In this way, the BdG Hamiltonian of this structure in basis $\psi =(\psi _{t\uparrow },\psi _{t\downarrow },\psi _{b\uparrow }, \psi _{b\downarrow },\psi ^*_{t\downarrow },-\psi ^*_{t\uparrow },\psi ^*_{b\downarrow },-\psi ^*_{b\uparrow })$ where the subscripts *t* and *b* refer to the top and bottom surfaces of a TI layer and $\uparrow \downarrow$ refers to spin, is obtained as follows^16^.

$$\begin{aligned} H_{\varvec{k}}=v_f \eta _z \tau _z (k_y \sigma _x- k_x \sigma _y) + m\eta _0\tau _0\sigma _z+\eta _z(m_k \tau _x -\tau _y t_z \sin k_z d )\sigma _0 +\varvec{\Delta }. \end{aligned}$$

Pauli matrices ${\varvec{\sigma }}$, ${\varvec{\tau }}$ and ${\varvec{\eta }}$ act in spin, top-bottom surfaces of TI layers and Nambu spaces, respectively and $m_k= t_s + t_D \cos (k_z d)$. The $8\times 8$ matrix $\varvec{\Delta }$ depend on the phase relationship of the top and bottom superconductors in each unit cell which consists of a TI layer and two SC layers surrounded it. If both superconductors have the same order parameters, $\Delta _0 e^{i\phi }$, then $\varvec{\Delta }=\Delta _0\eta _x \tau _0\sigma _0$ which is diagonal in $\sigma$ and $\tau$ spaces and therefore the realized Weyl superconductor has the scalar pairing^9^. But, If top-bottom superconductors have a $\pi$ phase difference, $\varvec{\Delta }=\Delta _0\eta _x \tau _z\sigma _0$, which changes sign upon inversion and is pseudoscalar under Lorentz transformation^16^.

Now, imagine setting the alternating phases to be $\Delta _0 e^{i(\phi +\chi )}$ and $\Delta _0 e^{i(\phi -\chi )}$ we obtain $\varvec{\Delta }=\Delta _0 e^{i\phi }\eta _x (\cos \chi \tau _0 +i\sin \chi \tau _z)\sigma _0$. This naturally combines both scalar and pseudo-scalar superconducting orders and is a way to achieve a proximitized realization of a Weyl superconductor with the most general s-wave order parameter $\Delta _s+i\Delta _5 \gamma ^5$ where $\Delta _s=\Delta _0 e^{i\phi } \cos \chi$ and $\Delta _5=\Delta _0 e^{i\phi } \sin \chi$. $\gamma ^5$ in this expression is identified as $\tau _z \sigma _0$ which is the same as $\gamma ^5$ in Weyl representation.

As pointed out in the original paper^15^, the superconductors in the alternating stack of SC/TI can be flux biased in a way to induce desired phase profile for the superconductors. This construction can be employed to design a situation where the phases of superconductors alternate between $\phi +\chi$ and $\phi -\chi$. From above construction, one produces a synthetic $\Delta _5$ that satisfies $\Delta _5/\Delta _s = \tan {\chi }$ where $\chi$ can be *tuned* by external flux bias as follows: Fig. 1 shows a schematic representation of the superconducting system, which consists of a multilayer structure of SC and TI layers. The superconductors are subject to a magnetic field ($\vec {B}$) that induces alternating fluxes $\pm \Phi /\Phi _0$ ($\Phi _0$ is the flux quantum) on the SCs surrounding the TI layer. Therefore $\chi$ can be identified as ${2\pi }\Phi /\Phi _0$.

We have assumed that the superconducting gap of each layer is independent and the magnetic flux threaded per ring is fully converted to the superconducting phase for each layer. This implies that the Josephson coupling between the layers is negligible. We justify this approximation by considering the following facts: (i): The superconductors used in our model are s-wave metal superconductors, which have a short coherence length. (ii) The magnetization of the TI layer causes the coherence lenght to be further reduced inside the TI layer. (iii) The TI layer acts as a barrier between top and bottom superconductors, which weakens the Josephson coupling between them. Therefore, we can ignore the effect of the intrinsic Josephson coupling on the flux dependence of the chiral angle $\chi$.

Raising $\chi$ from zero to $2\pi$ which corresponds to raising $\Phi$ from zero to ${\Phi _0}$, covers the entire angular span of the complex plane $(\Delta _s,\Delta _5)$. At $\chi =0$ where all the superconductors have the same phase, we have a conventional Weyl superconductor corresponding to $(\Delta _s=\Delta _0,\Delta _5=0)$ in the complex plane of $(\Delta _s,\Delta _5)$. At $\chi =\pi /2$ we have pure pseudoscalar superconductivity $(\Delta _s=0,\Delta _5=\Delta _0)$. At $\chi =\pi$ we have $(\Delta _s=-\Delta _0,\Delta _5=0)$, and so on and so forth. Between the above limits when $\chi$ has a generic value, we will have a generic s-wave Weyl superconductor with order parameter $\Delta _s+i\Delta _5\gamma ^5$. We note that an overall phase $e^{i\phi }$ does not affect our arguments. In other words, $\Delta _0$ could be complex and then factorizing the common phase factor of $\Delta _s$ and $\Delta _5$, the order parameter will be $e^{i\phi } (\Delta _s+i\Delta _5\gamma ^5)$.

**Figure 2:**
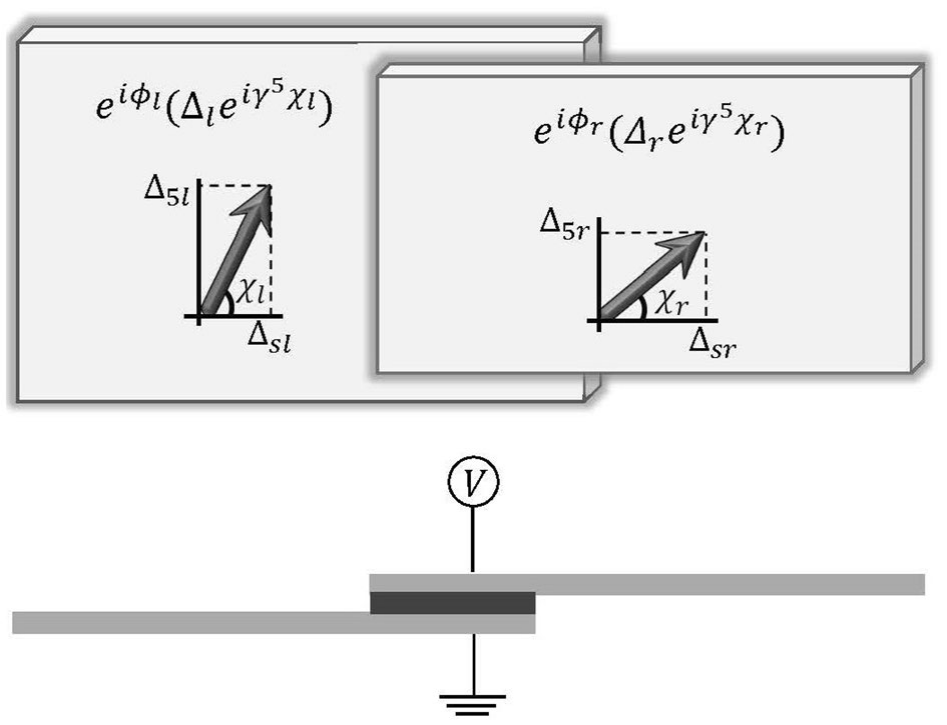
Top panel shows schematic top-view of the S|N|S junction. The lower panel shows the side view. Each of $a=r/l$ (left/right) superconductors, the superconductivity is specified by an strength $\Delta$ and *two phase angles* $(\phi ,\chi )$. The Argand diagram in an emergent complex plane of $(\Delta _s,\Delta _5)$ defines the chiral angle $\chi$ as a new degree of freedom in superconducting Weyl semimetals.

### Properties of the $(\Delta _s,\Delta _5)$ superconductivity

The general pairing potential $e^{i\phi }(\Delta _s+i\Delta _5\gamma ^5)$, manifestly breaks the standard (charge) *U*(1) symmetry. But under chiral gauge transformation $\Psi _e\rightarrow e^{i\gamma ^5\theta /2}\Psi _e$ where the $\gamma ^5$ is $\textrm{diag}(1,1,-1,-1)$^4,17^, we have, $\bar{\Psi }_e \rightarrow \bar{\Psi }_e e^{i\gamma ^5\theta /2}$ and $\Psi _h\rightarrow e^{-i\gamma ^5\theta /2}\Psi _h$. Therefore the pairing preserves the axial *U*(1) symmetry in the Fermion sector.

The diagonal elements of $\gamma ^5$ are the chirality eigenvalues and for real $\Delta _s$ and $\Delta _5$, the prefactor *i* guarantees the Hermiticity of the resulting Weyl-Bogoliubov-De Gennes (WBdG) Hamiltonian. Therefore the amplitude $\Delta _5$ denotes nothing but the previously found *pseudoscalar* superconductivity^12^. Such form of pseudoscalar superconductivity, spontaneously breaks not only the global *U*(1) symmetry (that is broken by every SC once a non-zero $\Delta _s$ is picked up), but also the parity symmetry that corresponds to realizing one of the eigenvalues of $\gamma ^5$ and giving the eigenvalues $e^{i\phi }(\Delta _s\pm i\Delta _5)$. Here choosing either of ± sings corresponds to spontaneously breaking a $Z_2$ symmetry. Hence, after factoring out an overall *U*(1) phase, the remaining $\Delta _s$ and $\Delta _5$ will be both real numbers. Now, consider the geometric algebra^18^ constructed from scalar $\mathbb {1}$ and the *pseudoscalar* $\gamma ^0\gamma ^1\gamma ^2\gamma ^3$ ($=-i\gamma ^5$) of the Clifford algebra: $\Delta _s {\mathbb 1}-\Delta _5\gamma ^0\gamma ^1\gamma ^2\gamma ^3$. This means apart from the *U*(1) phase $e^{i\phi }$, the most generic form of superconductivity in WSMs can be represented by a number in the complex plane $(\Delta _s,\Delta _5)$. The conventional superconductors are confined to the real axis of this plane, while the pure pseudoscalar superconductors^12,19^ are confined to the imaginary axis of this plane.

In order to appreciate the importance of the notion of the complex $(\Delta _s,\Delta _5)$ plane, let us consider a purely pseudoscalar superconductor^19^, $(0,\Delta _5)$. The complex plane structure allows to immediately understand the topologically non-trivial structure of such a superconductor. The coefficient “i”$=e^{i\pi /2}$ in front of $\Delta _5$, directly enters the amplitude of Andreev reflection at a superconductor-normal interface. This amounts to an additional phase change of $\pi /2$ upon every Andreev reflection (for more details refer to supplementary material). Therefore in a superconductor-normal-superconductor (S|N|S) Josephson junction, a total phase of $\pi$ is accumulated at the two interfaces of the S|N|S junction. Such a $\pi$ phase corresponds to change in the number parity. Therefore in a closed loop geometry of a Josephson junction, the electron has to traverse the loop once again, giving rise to the $4\pi$ (two rounds) periodic Josephson effect. The $4\pi$-periodic Josephson effect is a hallmark of topological superconductivity and its associated Majorana modes^19^. This can be the most natural explanation for the observed $4\pi -$periodic Andreev bound states in such systems^20^. So the Josephson physics on the real axis ($\Delta _s$) and imaginary axis ($\Delta _5$) are significantly different. Several papers have investigated the modified Josephson effect in different systems that supports Majorana bound states, such as quantum wires^21–23^ and semiconductor-superconductor 1D heterostructures^24^. Now we are going to explore the *chiral* Josephson current and a remarkably rich physics of half-vortices in Josephson arrays of Weyl superconductors with $(\Delta _s,\Delta _5)$ superconductivity.

### Josephson coupling

So far, We have shown how a Weyl superconductor with both scalar and pseudoscalar order parameters can be realized. In the following sections, we will consider the most general form of spin-singlet superconductivity for a Weyl superconductor and calculate the normal and chiral Josephson currents in Josephson junctions composed of these superconductors. Alternative way of expressing the complex algebra $(\Delta _s,\Delta _5)$ of superconducting Dirac/Weyl materials is to specify it by an strength $\Delta$ and two angles $(\phi ,\chi )$. $\phi$ being the *U*(1) phase couples to external EM fields, while $\chi$ is the polar angle in the complex plane $(\Delta _s,\Delta _5)$. This way pairing equation can be represented as

1 $$\begin{aligned} e^{i\phi }(\Delta _s\mathbb {1}+i\Delta _5\gamma ^5)=e^{i\phi }\Delta e^{i\gamma ^5\chi }. \end{aligned}$$

where $\Delta =\sqrt{\Delta _s^2+\Delta _5^2}$ is the amplitude of superconducting order parameter and the chiral angle (CA) is defined by $\tan \chi =|\Delta _5/\Delta _s|$. Therefore we need to extend the Josephson physics that involves a single phase $\phi$^25^, to include now a pair of phase variables $(\phi ,\chi )$.

Given two superconductors $\Delta _a e^{i\phi _a} e^{i\chi _a}$ where $a=l,r$ corresponds to left/right superconductor as in Fig. 2, how will the CAs, $\chi _a$ modify the Josephson effect? The answer to this fundamental question will provide us with the effective Hamiltonian governing the dynamics of phase fields $(\phi ,\chi )$ in Josephson arrays. The chemical potential difference between the left (*l*) and right (*r*) superconductors is set by the voltage *V* across the barrier. We assume that the barrier layer is sufficiently thin for electrons to tunnel through, and that the tunneling process can be regarded as a small perturbation. The tunneling current is given by^25^

2 $$\begin{aligned} I = \dfrac{e}{\hbar ^2} \dfrac{1}{v_l v_r} \sum _{{\varvec{k}},{\varvec{q}}} \int _{-\infty }^t dt' ~ e^{0_+ t'} Tr\bigg [\langle \hat{{\varvec{c}}}_{\varvec{k}}(t) \hat{{\varvec{c}}}_{\varvec{k}}^\dagger (t') \rangle \hat{T}_{{\varvec{k}}{\varvec{q}}} \eta _z \langle \hat{{\varvec{d}}}_{\varvec{q}}^* (t) \hat{{\varvec{d}}}_{\varvec{q}}^T(t') \rangle ^T \hat{T}_{{\varvec{q}}{\varvec{k}}} - \langle \hat{{\varvec{c}}}_{\varvec{k}}^* (t') \hat{{\varvec{c}}}_{\varvec{k}}^T(t) \rangle ^T \hat{T}_{{\varvec{k}}{\varvec{q}}} \eta _z \langle \hat{{\varvec{d}}}_{\varvec{q}}(t') \hat{{\varvec{d}}}_{\varvec{q}}^\dagger (t) \rangle \hat{T}_{{\varvec{q}}{\varvec{k}}}\bigg ], \end{aligned}$$

where $v_l (v_r)$ is the volume of the *l* (*r*) superconductor, $\hat{{\varvec{c}}}_{\varvec{k}}$ and $\hat{{\varvec{d}}}_{\varvec{q}}$ are the electron field operators that annihilate in *l* and *r* superconductors at wave vectors ${\varvec{k}}$ and ${\varvec{q}}$, respectively and $\hat{T}_{{\varvec{k}}{\varvec{q}}}$ is the tunneling matrix element between them. We further assume that the tunneling is independent of spin and chirality and does not break the time-reversal symmetry, $\hat{T}_{{\varvec{k}}{\varvec{q}}}=\hat{T}^*_{-{\varvec{q}},-{\varvec{k}}}$. Therefore the tunneling matrix in the chirality, spin and Nambu space is specified by the unit matrices, $\tau _0$, $\sigma _0$ and $\eta _0$, respectively: $\hat{T}_{{\varvec{k}}{\varvec{q}}}=t \sigma _0 \tau _0 \eta _0$. ${\varvec{\sigma }}$, ${\varvec{\tau }}$ and ${\varvec{\eta }}$ are Pauli matrices act in spin, chirality(pseudo-spin corresponds to top-bottom layer of periodic TI-SC multilayer^26^) and Nambu space, respectively (The details are available in the supplementary file).

In situations where some. form of boundary conditions^27–30^ impose a chirality reversal during the tunneling, one has to make a replacement $\tau _0\rightarrow \tau _z$, where $\tau _z$ is the third Pauli matrix in the chirality space. We find that the later form of tunneling process does not change our main result. Hence, in what follows we focus on the simplest case given above. The factor $e^{0_+t'}$, guarantees that the integrand vanishes for $t' \rightarrow -\infty$ and thereby ensures the convergence of the $t'$ integral.

It is straightforward to calculate the trace terms in current formula (2) by evaluating the expectation values as functions of *V*. It has two parts. One is the single particle part which is not relevant to Josephson current. Here we only focus on the second term that describes the transport of the Cooper pairs which includes the following term,

3 $$\begin{aligned} Tr\big [\langle \hat{{\varvec{c}}}_{\varvec{k}}(t) \hat{{\varvec{c}}}_{\varvec{k}}^\dagger (t') \rangle \hat{T}_{{\varvec{k}}{\varvec{q}}} \eta _z \langle \hat{{\varvec{d}}}_{\varvec{q}}^* (t) \hat{{\varvec{d}}}_{\varvec{q}}^T(t') \rangle ^T \hat{T}_{{\varvec{q}}{\varvec{k}}}\big ] \supset e^{ieV(t+t')/\hbar } f(\varepsilon _{\varvec{k}},\varepsilon _{\varvec{q}},t) - e^{- ieV(t+t')/\hbar } f^*(\varepsilon _{\varvec{k}},\varepsilon _{\varvec{q}},-t), \end{aligned}$$

where $f(\varepsilon _{\varvec{k}},\varepsilon _{\varvec{q}},t)$ is defined as

4 $$\begin{aligned} \dfrac{\Delta _l \Delta _r}{\varepsilon _{\varvec{k}}\varepsilon _{\varvec{q}}} n_-(\varepsilon _{\varvec{k}}) n_-(\varepsilon _{\varvec{q}}) e^{i( \phi _l - \phi _r)} \cos (\chi _l - \chi _r). \end{aligned}$$

Here $\varepsilon _{{\varvec{k}}}=\sqrt{k_x^2+k_y^2+k_z^2+\Delta _{l}^2}$ is the dispersion relation for the excitations of the left superconductor. Similarly the $\varepsilon _{{\varvec{q}}}$ which is obtained by $k\rightarrow q$ and $\Delta _l\rightarrow \Delta _r$ is the dispersion relation of the right superconductor. Furthermore, $n_\pm (\varepsilon _{{\varvec{k}}/{\varvec{q}}}) = n(\varepsilon _{{\varvec{k}}/{\varvec{q}}}) e^{i \varepsilon _{{\varvec{k}}/{\varvec{q}}} t/ \hbar } \pm n(-\varepsilon _{{\varvec{k}}/{\varvec{q}}}) e^{-i \varepsilon _{{\varvec{k}}/{\varvec{q}}} t/\hbar }$ where $n(\varepsilon _{{\varvec{k}}/{\varvec{q}}})=(e^{\beta \varepsilon _{{\varvec{k}}/{\varvec{q}}}}+1)^{-1}$ is the Fermi-Dirac distribution function. There are some other terms in Eq. (3) which include $(k_x \sigma _x + k_y \sigma _y)$ and $(k_x\sigma _y - k_y\sigma _x)$ but these terms give null contributions upon integration over $k_x$ and $k_y$, so we drop them. As it is seen in *f*, the dependence on the CA $\chi$ is of the $\cos (\chi _l-\chi _r)$ form. This result is robust against variations in the boundary conditions (i.e. replacing $\tau _0\rightarrow \tau _z$ in the tunneling matrix).

### Half-vortices

Substitution of  (3) in (2), the total Josephson current becomes,

5 $$\begin{aligned} I = I_{s} \cos (\chi _l-\chi _r), \end{aligned}$$

where $I_{s}$ is the standard form of the AC Josephson current with frequency 2*eV*/*h* that also appears in conventional superconductors,

6 $$\begin{aligned} I_{s}=I_{ss} \sin (\dfrac{2eVt}{\hbar }+\phi _l-\phi _r)+ I_{sc} \cos (\dfrac{2eVt}{\hbar }+\phi _l-\phi _r). \end{aligned}$$

Here $I_{ss}$ and $I_{sc}$ are functions of $\Delta _l$ and $\Delta _r$ such that at zero voltage the $I_{sc}$ becomes zero but $I_{ss}$ has a finite value and determines the critical current. For more details we refer the readers to the supplementary material. Let us now focus at $V=0$ where $I_{sc}$ becomes zero and the second term disappears. In the absence of deriving voltage, the $I_s$ solely arises from the phase difference $\phi _l-\phi _r$ of the superconductors:

7 $$\begin{aligned} I_s=I_{ss} \sin (\phi _l-\phi _r)\cos (\chi _l-\chi _r). \end{aligned}$$

Generalization of a single Josephson junction into an array of Josephson junctions on a lattice whose sites are labeled by *i*, *j*, yields

8 $$\begin{aligned} I_{ij}=I_{ss} [\sin (\phi _{ij}+\chi _{ij})+\sin (\phi _{ij}-\chi _{ij})]/2, \end{aligned}$$

where $\phi _{ij}=\phi _i-\phi _j$ and $\chi _{ij}=\chi _i-\chi _j$. The above two terms are indeed very suggestive: if one defines new phase fields $\varphi ^\pm _{ij}=\phi _{ij}\pm \chi _{ij}$^31^, it can be interpreted as an ordinary Josephson current for the right/left phase variables $\varphi ^\pm$. In the continuum, the $\phi$ couples to EM gauge field via $\partial _\mu \phi \rightarrow \partial _\mu \phi -(2e)A$. The above two terms in the current, when integrated with respect to the phase difference $\phi _{ij}$ to obtain the energy, will produce $-\cos (\phi _{ij}+\chi _{ij})-\cos (\phi _{ij}-\chi _{ij})$. Therefore the classical energy of the Josephson array will be

9 $$\begin{aligned} E[{\phi _i,\chi _i}]= - \lambda _J \sum _{\langle ij\rangle } \cos (\phi _{ij})\cos (\chi _{ij}). \end{aligned}$$

**Figure 3:**
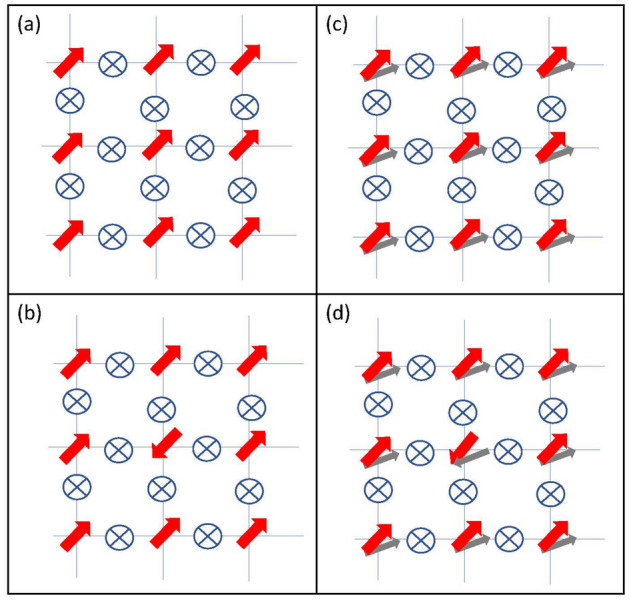
(**a**,**b**) represent the lowest energy state and an excited state of a 2D Josephson array of scalar superconductors, $\Delta _s$. Red arrows represent the phase variables $\phi _i$ and $\otimes$s are the Josephson junctions. In (**c**,**d**) the chiral angles are also important and are denoted by gray arrows. One possible ground state for $\phi _i$ (red arrows). In this case, the CA phases also point in their own fixed direction. (**d**) The phase (red) of the second superconductor (counting from the left) is flipped. This effect can be compensated by an associated flip in the $\chi$.

This equation is a generalization of the standard Josephson potential energy $-\sum _{\langle i,j\rangle }\cos {\phi _{ij}}$ (the classical XY model) which is minimized when all the superconductors of the Josephson array, have the same phase, namely $\phi _{ij}=0$. The ground state of the standard XY model is depicted by the red arrows in Fig. 3a. Every out of phase junction has the energy cost $2\lambda _J$. For example the central superconductor in Fig. 3b is completely out of phase and costs $2\times 2\lambda _J$. For simplicity let us focus on the continuum limit of the Josephson array. At low temperatures, the partition function of classical XY model is dominated by slowly varying configurations of the field $\phi ({\varvec{r}})$, and the $\cos \phi$ term will become $|\nabla \phi |^2$ to penalize deviations from the uniform *U*(1) phase configurations^32,33^. The configurations in the excited states of this model for which the identification $\phi \sim \phi \pm 2\pi$ round a circle in the real space can be made, correspond to vortex/anti-vortex solutions.

Now let us turn our attention to Fig. 3c,d where in addition to the *U*(1) phase variables (red arrows), the CA variables (gray arrows) also enter the game as a (pseudoscalar) background field. The important feature of Eq. (9) is a locking between the rotor variable $\phi$ of the XY model and background field $\chi$. While in the XY model, every phase (red) shift $\phi \rightarrow \phi +\pi$ such as the one in Fig. 3b entails an energy cost of $2\lambda _J$, in the extended XY model of Eq. (9) this can be compensated by a corresponding flip in the chiral angle field (gray) $\chi$ of the two Weyl superconductors. While Fig. 3c corresponds to the ground state in a uniform background $\chi$-field, by modifying the configurations of the background pseudoscalar $\chi$-field one may access a ground state such as Fig. 3d. The same phase flip can be engineered for any other superconductor. This situation bears certain similarity to half-vortices in superfluid $^3$He-A where the matrix of order parameter of superfluid is given by the product of its spin and orbital parts^34^, then a change of sign of orbital part of the order parameter acquired over any closed path in the liquid corresponding to half quantum vortex can be compensated by the change of sign of the spin part of the order parameter, so that the whole order parameter will be single-valued. But the present scenario differs from the above example in that here the pseudoscalar field $\chi$ can be *externally tuned* to a synthetic half-vortex configuration. Such a engineered half-vortex of $\chi$ will bind a half-vortex of $\phi$ due to the locking in Eq. (9). This can be thought of trapping a $\phi$-half-vortex. The fact that topologically non-trivial configurations of the background field $\chi$ can bind a $\phi$-half-vortex is quite similar to the way in which the vortex core (topologically non-trivial configuration) of a p-wave superconductor binds Majorana fermions^35^. If there one produces another $\chi$-half-vortex, it can bind the other $\phi$-half-vortex. Otherwise the other $\phi$-half-vortex can only bind to the boundary of the system.

Eq. (9) is a novel classical statistical mechanics problem that deserves investigation of its own. Nevertheless a qualitative physics of this model is evident from the following consideration. The Villain expansion of the ordinary XY model consists in representing the partition function of a $\cos \theta$ interaction featuring minima at $2\pi n$ in terms of parabolas centered around these minima. Let us now think of a completely different configuration of the $\chi$-field, namely a staggered configuration where it alternates its sign, one can generate new set of minima for $\cos \phi$ that are located at half-integer multiples of $2\pi$. In this way, the staggered pseudoscalar field $\chi$ gives rise to a set of parabolic minima located at integer and half-integer multiples of $2\pi$. One can imagine a similar Villain expansion around all these minima. The simplest function that in addition to minima at integer multiples of $2\pi$ also contains minima at half-integer multiples of $2\pi$ is achieved by an additional $\cos 2\phi$ term. In this case the natural *excitations* of the $\phi$-field will be half-vortices. On the other hand, uniform configuration of the background $\chi$ will favor full vortices that arise from the usual $\cos \phi$ term. Therefore, a generic configuration of $\chi$ can be modelled by a competition between $\cos \phi$ and $\cos 2\phi$^36^. Within this model, the superfluid phase of the extended XY model as a condensate of the *dual boson pairs*^36^, is a generalization of the XY model which has been introduced in various fields and studied by several authors (^37^ and references in it). The extended XY model is often used to describe two-band superconductors^38–42^. In those systems, the phase difference between the s-wave order parameters is either 0 or $\pi$. However, the difference between a generic Weyl superconductor and $s_{++}$ or $s_{+-}$ superconductors is that the two order parameters in the Weyl superconductor have a nontrivial phase difference that is neither 0 nor $\pi$, and that can be tuned by external flux bias.

The half vortices in a synthetic background $\chi$ field can be interpreted in terms of chirality imbalance in the following sense. Imagine a 2D array of Josephson junctions. In the absence of $\Delta _5$, a configuration of red arrows that round a closed path is identified as $\phi \sim \phi \pm 2 \pi$ defines a vortex/anti-vortex (excited) state. As discussed above, when both $\Delta _s$ and $\Delta _5$ are present, the superconducting phase round a circle can also be identified as $\phi \sim \phi \pm \pi$, which is accompanied by a corresponding (Ising) half-vortex in $\chi \sim \chi \mp \pi$. Since a half-vortex in $\phi$ is always accompanied by a half-vortex in $\chi$, and that $\varphi _\pm =\phi \pm \chi$, the half-vortex state can be alternatively interpreted as chirality polarized vortex state where the population of vortices in $\varphi _+$ and $\varphi _-$ fields differs.

Irrespective of our interpretation, the EM field only couples to $\phi$ and not to $\chi$. This will then provide a very sharp and definite experimental signature for the half-vortex state as follows: Straightforwardly following the arguments of Weinberg^43^, instead of obtaining the flux quantum $2\pi \hbar /(2e)=h/(2e)$ – as in ordinary superconductors – one finds the flux quantum $\pi \hbar /(2e)$. Interpreting this quantization rule as $h/(2{\tilde{e}})$ as in conventional superconductors, one is lead to conclude that $2{\tilde{e}}=4e$. This “doubling” can be immediately detected in the ac Josephson frequency of a single junction. Although it appears that we are dealing with a condensate of a *pair of Cooper pairs* (remember the boson doubling contained in $\phi \rightarrow 2\phi$), but in fact the root cause of this effect is the presence of the CA $\chi$ that plays a compensating role in Eq. (9). Such a frequency doubling can be regarded as manifestation of the staggered configuration of the $\chi$ field.

### Chiral Josephson current

So far, the chiral angle $\chi$ has lead to the formation of half-vortices and the associated confinement transition that separates this phase from the full vortex state. In a similar way that spatial variations of $\phi$ in a conventional superconductor leads to the conventional supercurrent, let us show that the spatial variations of $\chi$ leads to a chiral Josephson current. According to TI/SC model, this situation could be realized by continuously varying the $\chi$ difference of neighbour Weyl superconductors of the array which is controlled by the phase difference of top and bottom superconductors of each cell. To compute the chiral Josephson current, we return again to a single Josephson junction setup of Fig. 2. The only generalization we need to perform in Eq. (2) is to insert an additional tensor product of Pauli matrices $\tau _z\eta _z$. The $\tau _z$ encodes the fact that right- and left-handed chiral fermions have to enter the chiral current with opposite signs. The $\eta _z$ encodes the fact that time reversal operation mapping the electrons and holes in the BdG equation indeed flips the chirality. Therefore,

$$\begin{aligned} I_{5}= & {} \dfrac{e}{\hbar ^2} \dfrac{1}{v_l v_r} \sum _{{\varvec{k}},{\varvec{q}}} \int _{-\infty }^t dt'~ e^{0_+ t'} Tr\bigg [ \langle \hat{{\varvec{c}}}_{\varvec{k}}(t) \hat{{\varvec{c}}}_{\varvec{k}}^\dagger (t') \rangle \hat{T}_{{\varvec{k}}{\varvec{q}}} \eta _z(\tau _z\eta _z) \langle \hat{{\varvec{d}}}_{\varvec{q}}^* (t) \hat{{\varvec{d}}}_{\varvec{q}}^T(t') \rangle ^T \hat{T}_{{\varvec{q}}{\varvec{k}}}\\{} & {} - \langle \hat{{\varvec{c}}}_{\varvec{k}}^* (t') \hat{{\varvec{c}}}_{\varvec{k}}^T(t) \rangle ^T \hat{T}_{{\varvec{k}}{\varvec{q}}} \eta _z(\tau _z\eta _z) \langle \hat{{\varvec{d}}}_{\varvec{q}}(t') \hat{{\varvec{d}}}_{\varvec{q}}^\dagger (t) \rangle \hat{T}_{{\varvec{q}}{\varvec{k}}} \bigg ]. \end{aligned}$$

This equation yields the chiral Josephson current when the applied voltage $V=0$ as:

10 $$\begin{aligned} I_{5} =I_{ss}\sin {(\phi _l-\phi _r)}\sin (\chi _l-\chi _r). \end{aligned}$$

Note that the pair $(I,I_5)$ are both determined by the quantity $I_{ss}$ introduced in Eq. (5). The only difference is that, *I* is proportional to $\cos (\chi _l-\chi _r)$, while $I_5$ is proportional to the $\sin (\chi _l-\chi _r)$. In both cases the phase difference $\phi _{lr}$ is needed to drive the current of Cooper pairs. When the CA difference $\chi _{lr}$ is non-zero, in addition to the electric current, a net chirality is also carried by the Cooper pairs. It is useful to view the pair of numbers $(I,I_5)$ in a complex plane and note that the modulus of this complex numbers will be $I_{ss}\sin (\phi _{lr})$ of non-chiral superconductors. Therefore, for a generic chiral angle difference $\chi _{lr}$ the Josephson current has both non-chiral (*I*) and chiral ($I_5$) “components”. In the special case where the difference in the chiral angles of the two superconductors is $\chi _{lr}=\pi /2$, the non-chiral Josephson current will be zero, and the entire Josephson current will become chiral (i.e. along the imaginary axis in the complex plane of *I* and $I_5$).

Taking the continuum limit of the above results immediately reveals that the spatial variations of the CA, $\chi$ generates chiral currents. In 1+1 dimensional spacetime the above chiral current acquires a nice interpretation as follows: The continuum limit of the Josephson lattice of superconductors of Weyl semimetals according to Eq. (10) will give $I_5\propto \chi (x+\delta x_a)-\chi (x)\propto \partial _a\chi$, where *a* denotes a spatial direction. Lorentz boosting this result gives, $I_{5,\mu }\propto \partial _\mu \chi$. In $1+1$ spacetime dimensions, using the fact that $\gamma ^\mu =\varepsilon ^{\mu \nu }\gamma _\nu \gamma ^5$, the above result immediately gives $I^\mu =\varepsilon ^{\mu \nu }\partial _\nu \chi$ which has a manifest Goldston-Wilczek current form^33,44,45^ and satisfies the conservation $\partial _\mu I^\mu =0$. This qualifies the chiral Josephson current in 1+1 dimension as a direct manifestation of Goldstone-Wilczek current.

## Discussion

The most generic form of a spin-singlet superconducting Weyl semimetal is specified by a single *U*(1) phase $\phi$ and a pair of real numbers $(\Delta _s,\Delta _5)$ that form a complex algebra whose polar angle defines a chiral angle $\chi$. This angle does not result from spontaneous breaking of chiral *U*(1) symmetry. It merely represents the polar angle in Argand diagram of an emergent complex plane $(\Delta _s,\Delta _5)$. In this paper, we have shown how to engineer such a $(\Delta _5,\Delta _s)$ superconductor by alternatively flux-biased arrangements of BCS superconductors and topological insulators. As such the chiral angle $\chi$ can be directly controlled by the amount of flux bias. Furthermore, a sign change of $\Delta _5\rightarrow -\Delta _5$ ($\chi \rightarrow \chi +\pi$) can be induced by replacing the flux bias in the left and right side of the building block shown in Fig. 1. In fact sign reversal under reflection is a defining property of a *pseudo-*scalar. This setup will provide a framework to *externally apply* a pseudoscalar background field $\chi$. In fact application of pseudo-fields is an interesting topics of its own. In Ref. ^46^ it has been suggested that the a circularly polarized light on Weyl semimetals acts as a pseudo-gauge field. In this respect, our model can be regarded as much simpler setup where pseudo-gauge fields of finite strength can be externally applied to a system.

Depending on the configuration of the background pseudoscalar field $\chi$, a variety of interesting effects can be produced. (i) When one synthesizes an isolated half-vortex in the $\chi$ field, it will bind a $\phi$-half-vortex. This is similar to the way a vortex core in a p-wave superconductor binds a Majorana fermion^35^. In the case of an isolated engineered half vortex in the background $\chi$-field, the other $\phi$-half-vortex will be localized in the boundary of the system. (ii) For a staggered configuration of background $\chi$ field the additional minima generated by the alternating sign of the $\cos \chi$ at half-integer multiples of $2\pi$ will re-arrange the ground state in a way that the $\phi$-half-vortices will be supported. In a Josephson array based on the $(\Delta _s,\Delta _5)$ building element, where the background field is neither uniform, not staggered, rather it could belong to a range of configurations in between, interesting competition between the tendency to support vortices versus tendency to support half-vortices will set in. A possible externally tuned phase transition between the full vortex and half vortex case will be a confinement-deconfinement phase transition. The vortex Nernst-effect that continues to be present at temperatures above the BKT transition has proven to be a reliable signature of vorticity^47^.

## Methods

We have used the algebra of Dirac matrices, Fierz identities and tunneling mechanism to calculate the Josephson current. The calculation methods are detailed in supplementary material.

### Supplementary Information

Supplementary Information.

## Data Availability

The data supporting the findings of this study are available within the article and Supplementary material.
