# Supplementary material for: Synthetic complex Weyl superconductors, chiral Josephson effect and synthetic half-vortices
Source: Sci Rep. 2023 Oct 20;13:17976. doi: 10.1038/s41598-023-44910-0 (PMC10589258; doi:10.1038/s41598-023-44910-0)
Supplement: Supplementary file 1 — Supplementary Information. [file 41598_2023_44910_MOESM1_ESM.pdf]

Supplementary material: Synthetic complex Weyl superconductors, chiral Josephson effect and synthetic half-vortices

ANDREEV REFLECTION FROM PURELY PSEUDOSCALAR SUPERCONDUCTOR

We calculate the Andreev reflection at the interface of a Dirac/Weyl semimetal with a pseudoscalar superconductor. We show that such a peculiar SN interface gives rise to an additional phase change of $\pi/2$ compared to scalar superconductivity. The general Hamiltonian is of the Dirac-Bogoliubov-de Genes type given by

$$H_W = \begin{pmatrix} H_e - \mu & \Delta \\ \Delta^\dagger & \mu - \mathcal{T} H_e \mathcal{T}^{-1} \end{pmatrix} \tag{S1}$$

where, $\hat{H}_e = v(\boldsymbol{p}.\boldsymbol{\sigma})\tau_3$ represents the Hamiltonian for massless Weyl electrons with v the Fermi velocity and $\boldsymbol{p} = -i\hbar\boldsymbol{\nabla}$. $\boldsymbol{\sigma} = (\sigma_1, \sigma_2, \sigma_3)$ are the Pauli matrices of spin space and $\boldsymbol{\tau} = (\tau_1, \tau_2, \tau_3)$ are the Pauli matrices of pseudospin(Weyl node) space. $\mathcal{T} = -i\sigma_2\tau_0\mathcal{K}$ is the time reversal operator, so $\mu - \mathcal{T} H_e \mathcal{T}^{-1} = \mu - i\hbar v\tau_3(\boldsymbol{\sigma}\cdot\boldsymbol{\nabla})$ with μ is the chemical potential of the whole system. The Hamiltonian acts on the Nambu spinor $\psi = (\psi_e, \psi_h)^T$ with $\psi_e = (\psi_{+\uparrow}, \psi_{+\downarrow}, \psi_{-\uparrow}, \psi_{-\downarrow})^T$ and $\psi_h = \mathcal{T}\psi_e = (-\psi^*_{+\downarrow}, \psi^*_{+\uparrow}, -\psi^*_{-\downarrow}, \psi^*_{-\uparrow})$. The positive $(+)$ and negative $(-)$ indices represent the chirality (Weyl cone) indices while up and down arrows denote the spin indices. In this Nambu basis, Δ stands for the superconducting pair potential. On the normal side we set it equal to $\Delta = 0$ but On the superconducting side we consider a s-wave order order parameter with $\Delta = (\Delta_s e^{i\phi})\sigma_0\tau_x$ for a scalar (conventional) superconductivity, and $\Delta = (\Delta_5 e^{i\phi})\sigma_0\tau_y$ for pseudoscalar superconductivity. These superconductivities couple the ψ_+ part of Nambu basis to ψ^*_- part and ψ_- part to ψ^*_+. As a result, to diagonalize the above block-diagonal Hamiltonian, we solve the two following independent eigen equations in which we set $\hbar = 1$ and $v = 1$;

$$\begin{pmatrix} \mp i\partial_z - \mu & \mp i(\partial_x - i\partial_y) & \Delta^{\uparrow\downarrow}_{\pm\mp} & \Delta^{\uparrow\uparrow}_{\pm\mp} \\ \mp i(\partial_x + i\partial_y) & \pm i\partial_z - \mu & \Delta^{\downarrow\downarrow}_{\pm\mp} & \Delta^{\downarrow\uparrow}_{\pm\mp} \\ \Delta^{\downarrow\uparrow *}_{\mp\pm} & \Delta^{\downarrow\downarrow *}_{\mp\pm} & \mu \pm i\partial_z & \pm i(\partial_x - i\partial_y) \\ \Delta^{\uparrow\uparrow *}_{\mp\pm} & \Delta^{\uparrow\downarrow *}_{\mp\pm} & \pm i(\partial_x + i\partial_y) & \mu \mp i\partial_z \end{pmatrix} \begin{pmatrix} \psi_{\pm\uparrow} \\ \psi_{\pm\downarrow} \\ -\psi^*_{\mp\downarrow} \\ \psi^*_{\mp\uparrow} \end{pmatrix} = \varepsilon \begin{pmatrix} \psi_{\pm\uparrow} \\ \psi_{\pm\downarrow} \\ -\psi^*_{\mp\downarrow} \\ \psi^*_{\mp\uparrow} \end{pmatrix}. \tag{S2}$$

If we assume the $x - y$ plane as the interface between normal($z < 0$) and superconductor($z > 0$) regions, the k_x and k_y remain good quantum numbers. Therefore the eigenstates are of the plane wave $(u_1, u_2, v_1, v_2) \times exp(ik_x x + ik_y y)$ form which their energy ε depends on the matrix elements of Δ. In the normal region, the pair potential vanishes and the electron and hole parts decouple. For a right-hand electron incoming to the interface from the left(normal) side we have

$$\begin{pmatrix} k_z - \mu & k_x - ik_y \\ k_x + ik_y & -k_z - \mu \end{pmatrix} \begin{pmatrix} u_1 \\ u_2 \end{pmatrix} = \varepsilon \begin{pmatrix} u_1 \\ u_2 \end{pmatrix} \tag{S3}$$

and for a left-hand hole reflecting from the interface into left(normal) side we have

$$\begin{pmatrix} \mu + k_z & -(k_x - ik_y) \\ -(k_x + ik_y) & \mu - k_z \end{pmatrix} \begin{pmatrix} v_1 \\ v_2 \end{pmatrix} = \varepsilon \begin{pmatrix} v_1 \\ v_2 \end{pmatrix},$$

The corresponding eigenstates for electrons and holes are,

$$\psi^+_e = \begin{pmatrix} e^{-i\gamma}\cos(\theta/2) \\ \sin(\theta/2) \\ 0 \\ 0 \end{pmatrix} e^{ik_z z} \quad , \quad \psi^-_h = \begin{pmatrix} 0 \\ 0 \\ -e^{-i\gamma}\cos(\theta'/2) \\ \sin(\theta'/2) \end{pmatrix} e^{-ik_z z}, \tag{S4}$$

where $\theta = \arccos[k_z/(\varepsilon_e + \mu)]$ is the polar electron's incidence angle with the z axis normal to the interface, while γ is the azimuthal angle of incidence (we have used ϕ for superconducting phase, so we use the unusual symbol γ for azimuthal angle). $\theta' = \arccos[k_z/(\varepsilon_h - \mu)]$ is the polar angle of the reflected hole. The interface is in $x - y$ plane so the azimutal angle of reflected holes are similar to them for incidence electrons. Defining $k = \sqrt{k_x^2 + k_y^2 + k_z^2}$, $\varepsilon_e = \pm k - \mu$ and $\varepsilon_h = \pm k + \mu$. The eigen states for a left-hand electron incoming to the interface and a right-hand hole reflecting from the interface are,

$$\psi^-_e = \begin{pmatrix} \sin(\theta/2) \\ -e^{i\gamma}\cos(\theta/2) \\ 0 \\ 0 \end{pmatrix} e^{ik_z z} \quad , \quad \psi^+_h = \begin{pmatrix} 0 \\ 0 \\ \sin(\theta'/2) \\ -e^{i\gamma}\cos(\theta'/2) \end{pmatrix} e^{-ik_z z}, \tag{S5}$$

For given ε_e and k_z, if $\mu \to \infty$ then $\theta \to 0$ and also $\theta' \to 0$. Hence, the spinors of incoming electrons become $\begin{pmatrix}1\\0\end{pmatrix} e^{ik_z z}$ and $\begin{pmatrix}0\\1\end{pmatrix} e^{ik_z z}$ and for reflecting holes become $\begin{pmatrix}1\\0\end{pmatrix} e^{-ik_z}$ and $\begin{pmatrix}0\\1\end{pmatrix} e^{-ik_z}$, respectively.

Now let us solve the Dirac/Weyl-BdG equation for the superconducting side where the coupling potential is non-zero. At first step, we consider a scalar superconductor on the top side ($z > 0$) of the NS junction. The corresponding BdG Hamiltonian is,

$$\begin{pmatrix} k_z - \mu & k_x - ik_y & \Delta_s e^{i\phi} & 0 \\ k_x + ik_y & -k_z - \mu & 0 & \Delta_s e^{i\phi} \\ \Delta_s e^{-i\phi} & 0 & \mu - k_z & -(k_x - ik_y) \\ 0 & \Delta_s e^{-i\phi} & -(k_x + ik_y) & \mu + k_z \end{pmatrix} \begin{pmatrix} u_1 \\ u_2 \\ v_1 \\ v_2 \end{pmatrix} = \varepsilon \begin{pmatrix} u_1 \\ u_2 \\ v_1 \\ v_2 \end{pmatrix}, \tag{S6}$$

which gives $\varepsilon_s = k \pm \sqrt{\Delta_s^2 + \mu^2}$ with subscript s stands for the "scalar". In the $\theta \to 0$ limit which corresponds to vertical incidences, the pair eigenstates are,

$$\psi_S^+ = \begin{pmatrix} e^{i\phi} \\ 0 \\ -e^{i\beta_s} \\ 0 \end{pmatrix} \quad , \quad \psi_S^- = \begin{pmatrix} 0 \\ e^{i\phi} \\ 0 \\ e^{-i\beta_s} \end{pmatrix}, \tag{S7}$$

with $\beta_s = \arccos\left[(k - \varepsilon_s)/\Delta_s\right]$. The superscripts $+$ and $-$ in above eigen functions refer to $\pm$ sign in the dispersion relation. It is straightforward to compute the Andreev reflection amplitude for electrons and holes which are given by,

$$r_e^a = -e^{-i\phi + i\beta_s}, \quad r_h^a = e^{2i\phi} r_e^a. \tag{S8}$$

To develop an intuition, one can simplify the corresponding expressions by taking the $\theta \to 0$ limit and considering retro Andreev reflection. In the following paragraphs we give details of this computation for pseudoscalar superconductivity that will manifest an extra factor "i", providing a hint that the pseudoscalar superconductivity acts like imaginary part of a more generic "complex" object as discussed in the main text.

Let us now focus on the pseudoscalar superconductivity for which the superconducting matrix is given by $\Delta = (i\Delta_5 e^{i\phi})\sigma_z$. The Dirac/Weyl-BdG equation in this case is,

$$\begin{pmatrix} k_z - \mu & k_x - ik_y & -i\Delta_5 e^{i\phi} & 0 \\ k_x + ik_y & -k_z - \mu & 0 & -i\Delta_5 e^{i\phi} \\ i\Delta_5 e^{-i\phi} & 0 & \mu - k_z & -(k_x - ik_y) \\ 0 & i\Delta_5 e^{-i\phi} & -(k_x + ik_y) & \mu - k_z \end{pmatrix} \begin{pmatrix} u_1 \\ u_2 \\ v_1 \\ v_2 \end{pmatrix} = \varepsilon \begin{pmatrix} u_1 \\ u_2 \\ v_1 \\ v_2 \end{pmatrix}. \tag{S9}$$

whose eigenvalues are $\varepsilon_{ps} = k \pm \sqrt{\Delta_5^2 + \mu^2}$. To bring out the essential physics of the imaginary "i", we only consider the vertical incidences with $k_x = k_y = 0$. In this case the eigenstats simplify to,

$$\psi_S^+ = \begin{pmatrix} 0 \\ e^{i\phi} \\ 0 \\ ie^{-i\beta_5} \end{pmatrix} \quad , \quad \psi_S^- = \begin{pmatrix} e^{i\phi} \\ 0 \\ ie^{i\beta_5} \\ 0 \end{pmatrix}. \tag{S10}$$

where $\beta_5 = \arccos\left[(k - \varepsilon)/\Delta_5\right]$. Comparing these eigenstates with those in Eq. (S7) for scalar superconductors, clearly shows an additional i prefactor in the hole part of the Nambu spinor which comes directly from i that appears in $i\Delta_5$ for pseudoscalar superconductivity.

Now we consider an incident electron from the normal WSM side (ψ_e^+) with energy $\varepsilon < \Delta_5$ and wave vector $\vec{k} = (k_x, k_y, k_z)$. Again because of the translational invariance, k_x and k_y are good quantum numbers, and so they do not change upon Andreev reflection. The k_z sign does however change in the reflection process. The incident electron would be reflected as a hole (ψ_h^-) through Andreev reflection channel and as an electron (ψ_e^-) through normal reflection channel. The amplitude of each reflection can be obtained from the continuity condition of the wave functions at the interface ($z = 0$). The wave functions at two sides of the interface can be expressed as,

$$\psi_N = \begin{pmatrix} e^{-i\gamma}\cos(\theta/2) \\ \sin(\theta/2) \\ 0 \\ 0 \end{pmatrix} + r \begin{pmatrix} -e^{-i\gamma}\cos(\theta/2) \\ \sin(\theta/2) \\ 0 \\ 0 \end{pmatrix} + r_a \begin{pmatrix} 0 \\ 0 \\ -e^{-i\gamma'}\cos(\theta'/2) \\ \sin(\theta'/2) \end{pmatrix}$$

$$\psi_S = a \begin{pmatrix} 0 \\ e^{i\phi} \\ 0 \\ ie^{-i\beta_5} \end{pmatrix} + b \begin{pmatrix} e^{i\phi} \\ 0 \\ ie^{-i\beta_5} \\ 0 \end{pmatrix} \tag{S11}$$

where r and r_a are the normal and Andreev reflection amplitudes, respectively. a and b are the coefficients of the quasiparticles in the superconductor region. θ' is the reflection angle and γ' is the azimuthal angle of the Andreev reflected hole while θ and γ are for incident electron. The continuity equations give,

$$\begin{cases} e^{-i\gamma}\cos(\theta/2) - re^{-i\gamma}\cos(\theta/2) = b\, e^{i\phi} \\ \sin(\theta/2) + r\sin(\theta/2) = a\, e^{i\phi} \\ -r_a e^{-i\gamma'}\cos(\theta'/2) = ib\, e^{-i\beta_5} \\ r_a\sin(\theta'/2) = ia\, e^{-i\beta_5} \end{cases} \Rightarrow \begin{cases} \dfrac{e^{-i\gamma}}{\tan(\theta/2)}(\dfrac{1-r}{1+r}) = \dfrac{b}{a} \\ \\ \dfrac{-e^{-i\gamma'}}{\tan(\theta'/2)} = \dfrac{b}{a} \end{cases},$$

which yield,

$$r = \frac{e^{-i(\gamma-\gamma')}\tan(\theta'/2) + \tan(\theta/2)}{e^{-i(\gamma-\gamma')}\tan(\theta'/2) - \tan(\theta/2)}. \tag{S12}$$

In the limit of retro-reflection where $\gamma = \gamma'$ and $\theta = \pi - \theta'$ so $\tan(\theta/2) = \cot(\theta'/2)$ one has,

$$r = \frac{1}{\cos(\theta)},$$
$$r_a = -i(2\cos\theta - 1)e^{-i(\phi-\beta_5)}. \tag{S13}$$

For vertical incidence with $\theta = 0$ the life becomes simpler and one has $r = 0$ and

$$r_a = -ie^{-i\phi+i\beta_5} = -e^{i\pi/2-i\phi+i\beta_5},$$
$$r'_a = e^{2i\phi}r_a, \tag{S14}$$

where r'_a is the Andreev reflection amplitude which has been obtained from similar equations for an incident hole, that can be either normal reflected as a hole or Andreev reflected as an electron.

Comparing (S14) and (S8) shows clearly that each electron-hole or hole-electron Andreev reflection at the interface of a normal Weyl/Dirac semimetal with a pseudoscalar superconductor, generates a $\pi/2$ phase change in addition to conventional phase changes occurring at the interface of a normal conductor with the standard scalar superconductor. It can be further seen that the additional i factor in r_a comes from the prefactor of i in our Nambu spinor which as we noticed below Eq. (S7), comes from Δ_5 and hence is tied with the pseudoscalar superconductivity and its parity breaking nature. This can be summarized in the jargon of A. Zee in his book[1], by stating that, nature Wick rotates the superconductivity (and Andreev reflections) when it breaks the parity symmetry.

CHIRAL TUNNELING CURRENT

As pointed out in the main text, the same way that separating two conventional (scalar) superconducting materials by a barrier gives rise to Josephson current, separating two pseudoscalar superconductors will give rise to a form of Josephson current that carries a net chirality, and hence the name chiral Josephson current or axial Josephson current can describe it. In this section we provide the detailed derivation of this phenomenon.

The basic formulation of this section is based on the textbook of Kita[2] that has been expanded for the case of Dirac/Weyl Hamiltonians. Consider two superconducting Weyl semimetals described by the most generic form of "complex superconducting order parameter in Eq. (2) of the main text. We further give subscript l and r to denote the superconducting WSM in the

left and right sides of the S|N|S junction as depicted in Fig. (1) of the main text. The superconducting orders are therefore, $\Delta_l = \Delta_l e^{i\phi_l} e^{i\chi_l}$ and $\Delta_r = \Delta_r e^{i\phi_r} e^{i\chi_r}$ where $\phi_{l,r}$ and $\chi_{l,r}$ are the the $U(1)$ and axial phases, respectively and $\Delta_{l/r}$ denotes the strength of the superconductivity for the left/right superconductor. The entire system may be described by the Hamiltonian,

$$H = H_l + H_r + T \tag{S15}$$

where H_l (H_r) is the Hamiltonian of the right (left) superconductor as described in (S1). We consider a potential difference between two superconductors, namely $\mu_l = \mu_r + eV$. To illustrate the basic physics, a weak tunneling is enough as it will allow a straightforward perturbative treatment. We therefore regard the tunneling Hamiltonian T as a perturbation which is given by,

$$T = \frac{1}{\sqrt{V_l V_r}} \sum_{\boldsymbol{kq}} \hat{\boldsymbol{c}}^\dagger_{\boldsymbol{k}} \hat{T}_{\boldsymbol{kq}} \sigma_z \hat{\boldsymbol{d}}_{\boldsymbol{q}}, \tag{S16}$$

where V_l (V_r) is the volume of the left (right) side. $\hat{\boldsymbol{c}}_{\boldsymbol{k}} = (\hat{c}_{\boldsymbol{k}+\uparrow}, \hat{c}_{\boldsymbol{k}+\downarrow}, \hat{c}_{\boldsymbol{k}-\uparrow}, \hat{c}_{\boldsymbol{k}-\downarrow}, \hat{c}^\dagger_{-\boldsymbol{k}+\uparrow}, \hat{c}^\dagger_{-\boldsymbol{k}+\downarrow}, \hat{c}^\dagger_{-\boldsymbol{k}-\uparrow}, \hat{c}^\dagger_{-\boldsymbol{k}-\downarrow})^T$ introduces the basis of field operators of the left side while $\hat{\boldsymbol{d}}_{\boldsymbol{q}} = (\hat{d}_{\boldsymbol{q}+\uparrow}, \hat{d}_{\boldsymbol{q}+\downarrow}, \hat{d}_{\boldsymbol{q}-\uparrow}, \hat{d}_{\boldsymbol{q}-\downarrow}, \hat{d}^\dagger_{-\boldsymbol{q}+\uparrow}, \hat{d}^\dagger_{-\boldsymbol{q}+\downarrow}, \hat{d}^\dagger_{-\boldsymbol{q}-\uparrow}, \hat{d}^\dagger_{-\boldsymbol{q}-\downarrow})^T$ is for the right side. $\hat{T}_{\boldsymbol{kq}} = t\sigma_0\tau_0\hat{\eta}_0$ is the tunneling matrix which can be assumed independent of spin, chirality and charge attributes. One can show that the phenomenon of axial/chiral Josephson current is robust against boundary conditions, and peculiar boundary conditions flipping the chirality ($\tau_0 \to \tau_z$) or spin ($\sigma_0 \to \sigma_z$), do not alter the main result.

The current operator can be expressed as the loss of positive charge on e.g. the left side and appearance of the same charge in the right side enabled by the tunneling process as follows:

$$\hat{I} = \frac{i}{\hbar} \frac{e}{V_l V_r} \sum_{\boldsymbol{k},\boldsymbol{q}} \hat{\boldsymbol{d}}^\dagger_{\boldsymbol{q}} \hat{T}_{\boldsymbol{qk}} \hat{\boldsymbol{c}}_{\boldsymbol{k}} \tag{S17}$$

where $\hat{T}_{\boldsymbol{qk}} = \hat{T}^*_{\boldsymbol{kq}}$ and the Hermitian nature of the above current operator is implicit in the definition of $\hat{\boldsymbol{c}}_{\boldsymbol{k}}$ that includes both creation and annihilation operators. According to linear response theory, when a perturbation such as tunneling is turned on, a non-equilibrium current is driven in the system. The equilibrium current is zero and hence for small T the current is,

$$I(t) = -\frac{i}{\hbar} \int_{-\infty}^{t} dt' \langle [\hat{I}(t), \hat{T}(t)] \rangle e^{0+t'}. \tag{S18}$$

where $\hat{I}(t) = e^{iH_0 t/\hbar} \hat{I} e^{-iH_0 t/\hbar}$ and $\hat{T}(t) = e^{iH_0 t/\hbar} \hat{T} e^{-iH_0 t/\hbar}$ with $H_0 = H_l + H_r$ being the unperturbed Hamiltonian. The factor $e^{0+t'}$ guarantees the convergence of the Fourier integral.

Using the definitions (S16) and (S17), and employing the Wick theorem, the current can be expressed as,

$$I(t) = \frac{e}{\hbar^2} \frac{1}{V_l V_r} \sum_{\boldsymbol{kq}} \int_{-\infty}^{t} dt' e^{0+t'} \, \mathrm{Tr} \Big[\langle \hat{\boldsymbol{c}}_{\boldsymbol{k}}(t) \hat{\boldsymbol{c}}^\dagger_{\boldsymbol{k}}(t') \rangle \hat{T}_{\boldsymbol{kq}} \sigma_z \langle \hat{\boldsymbol{d}}^*_{\boldsymbol{q}}(t) \hat{\boldsymbol{d}}^T_{\boldsymbol{q}}(t') \rangle^T \hat{T}_{\boldsymbol{qk}} - \langle \hat{\boldsymbol{c}}^*_{\boldsymbol{k}}(t') \hat{\boldsymbol{c}}^T_{\boldsymbol{k}}(t) \rangle^T \hat{T}_{\boldsymbol{kq}} \sigma_z \langle \hat{\boldsymbol{d}}_{\boldsymbol{q}}(t') \hat{\boldsymbol{d}}^\dagger_{\boldsymbol{q}}(t) \rangle \hat{T}_{\boldsymbol{qk}} \Big], \tag{S19}$$

where $\hat{\boldsymbol{c}}_{\boldsymbol{k}}(t) = e^{iH_l t/\hbar} \, \hat{\boldsymbol{c}}_{\boldsymbol{k}} \, e^{-iH_l t/\hbar}$ and $\hat{\boldsymbol{d}}_{\boldsymbol{q}}(t) = e^{iH_r t/\hbar} \, \hat{\boldsymbol{d}}_{\boldsymbol{q}} \, e^{-iH_r t/\hbar}$, and $\hat{\boldsymbol{d}}_{\boldsymbol{q}} = (\hat{\boldsymbol{d}}^\dagger_{\boldsymbol{q}})^T$. It is necessary to note that the Hamiltonian H_l of the left side of the system is a function of V, meaning that $H_l(V) = H_l(V=0) - V\hat{Q}_l$, where $\hat{Q}_l = e\sum_{\boldsymbol{k}\alpha\lambda} \hat{c}^\dagger_{\boldsymbol{k}\alpha\lambda} \hat{c}_{\boldsymbol{k}\alpha\lambda}$ with indices $\alpha = \uparrow, \downarrow$ and $\lambda = \pm$ denoting spin and chirality, respectively. Then the field operators $\hat{\boldsymbol{c}}_{\boldsymbol{k}}(t)$ thereby gains an extra time-evolution phase (proportional to the driving voltage V) with respect to $\hat{\boldsymbol{d}}_{\boldsymbol{q}}(t)$. Substituting this extra phase, we can express the current as

$$I = \frac{e}{\hbar^2} \frac{|t|^2}{V_l V_r} \sum_{\boldsymbol{k},\boldsymbol{q}} \int_{-\infty}^{t} dt' \, \mathrm{Tr} \left[\hat{\Gamma}(t) \langle \hat{\boldsymbol{c}}_{\boldsymbol{k}}(t) \hat{\boldsymbol{c}}^\dagger_{\boldsymbol{k}}(t') \rangle \hat{\Gamma}^*(t') \eta_z \langle \hat{\boldsymbol{d}}^*_{\boldsymbol{q}}(t) \hat{\boldsymbol{d}}^T_{\boldsymbol{q}}(t') \rangle^T - \hat{\Gamma}(t) \langle \hat{\boldsymbol{c}}^*_{\boldsymbol{k}}(t') \hat{\boldsymbol{c}}^T_{\boldsymbol{k}}(t) \rangle^T \hat{\Gamma}^*(t') \eta_z \langle \hat{\boldsymbol{d}}_{\boldsymbol{q}}(t') \hat{\boldsymbol{d}}^\dagger_{\boldsymbol{q}}(t) \rangle \right] e^{0+t'}, \tag{S20}$$

where all the expectations should be calculated at the unperturbed ground state corresponding to $V = 0$ and $\hat{\Gamma}(t)$ incorporates the extra phase described above,

$$\hat{\Gamma}(t) = \begin{pmatrix} e^{ieVt/\hbar} \sigma_0 \tau_0 & 0 \\ 0 & e^{-ieVt/\hbar} \sigma_0 \tau_0 \end{pmatrix}. \tag{S21}$$

To calculate expectation values needed in Eq. (S20), we expand $\hat{\boldsymbol{c}}_{\boldsymbol{k}}$ and $\hat{\boldsymbol{d}}_{\boldsymbol{q}}$ in terms of quasiparticle fields with relevant unitary matrices. Then we can express our results in terms of the occupation numbers $n(\varepsilon_{\boldsymbol{k}/\boldsymbol{q}}) = \sum_{\boldsymbol{k}/\boldsymbol{q}\alpha\lambda} \langle \gamma^\dagger_{\boldsymbol{k}\alpha\lambda} \hat{\gamma}_{\boldsymbol{k}/\boldsymbol{q}\alpha\lambda} \rangle$.

For each superconductor the explicit matrix form of Dirac/Weyl-BdG Hamiltonian is,

$$H=\begin{pmatrix} k_z^+-\mu^+ & k_x^+-ik_y^+ & 0 & 0 & \Delta e^{i(\phi-\chi)} & 0 & 0 & 0\\ k_x^++ik_y^+ & -k_z^+-\mu^+ & 0 & 0 & 0 & \Delta e^{i(\phi-\chi)} & 0 & 0\\ 0 & 0 & -k_z^--\mu^- & -(k_x^--ik_y^-) & 0 & 0 & \Delta e^{i(\phi+\chi)} & 0\\ 0 & 0 & -(k_x^-+ik_y^-) & k_z^--\mu^- & 0 & 0 & 0 & \Delta e^{i(\phi+\chi)}\\ \Delta e^{-i(\phi-\chi)} & 0 & 0 & 0 & \mu^--k_z^- & -(k_x^--ik_y^-) & 0 & 0\\ 0 & \Delta e^{-i(\phi-\chi)} & 0 & 0 & -(k_x^-+ik_y^-) & \mu^-+k_z^- & 0 & 0\\ 0 & 0 & \Delta e^{-i(\phi+\chi)} & 0 & 0 & 0 & \mu^++k_z^+ & k_x^+-ik_y^+\\ 0 & 0 & 0 & \Delta e^{-i(\phi+\chi)} & 0 & 0 & k_x^++ik_y^+ & \mu^+-k_z^+ \end{pmatrix}, \tag{S22}$$

which is written in the basis,

$$\Psi=(\psi_{+\uparrow},\psi_{+\downarrow},\psi_{-\uparrow},\psi_{-\downarrow},-\psi^*_{-\downarrow},\psi^*_{-\uparrow},-\psi^*_{+\downarrow},\psi^*_{+\uparrow})^T=(\Psi_e,\Psi_h)^T. \tag{S23}$$

$\boldsymbol{k}^\pm=\boldsymbol{k}\pm\boldsymbol{b}$ where $\boldsymbol{b}$ is the momentum splitting of the Weyl nodes. $\mu^\pm$ are the chemical potential which are different in the most general form and $\mu^+-\mu^-$ is the energy splitting of the Weyl nodes. Diagonalizing the Hamiltonian, gives $\pm\varepsilon_{\boldsymbol{k}^\pm}$ where

$$\varepsilon_{\boldsymbol{k}^\pm}=\sqrt{(k^\pm)^2+\Delta^2}-\mu^\pm, \tag{S24}$$

where, regardless of the plane wave prefactor, the eigenvectors corresponding to $+\varepsilon_{\boldsymbol{k}^\pm}$ are,

$$\begin{pmatrix} \sqrt{\frac{\xi_{\boldsymbol{k}^+}+k_z^+}{2\xi_{\boldsymbol{k}^+}}}\\ \frac{k_x^++ik_y^+}{\sqrt{2\xi_{\boldsymbol{k}^+}(\xi_{\boldsymbol{k}^+}+k_z^+)}}\\ 0\\ 0\\ \frac{\Delta e^{-i(\phi-\chi)}}{\sqrt{2\xi_{\boldsymbol{k}^+}(\xi_{\boldsymbol{k}^+}+k_z^+)}}\\ 0\\ 0\\ 0 \end{pmatrix},\quad \begin{pmatrix} \frac{k_x^+-ik_y^+}{\sqrt{2\xi_{\boldsymbol{k}^+}(\xi_{\boldsymbol{k}^+}-k_z^+)}}\\ \sqrt{\frac{\xi_{\boldsymbol{k}^+}-k_z^+}{2\xi_{\boldsymbol{k}^+}}}\\ 0\\ 0\\ 0\\ \frac{\Delta e^{-i(\phi-\chi)}}{\sqrt{2\xi_{\boldsymbol{k}^+}(\xi_{\boldsymbol{k}^+}-k_z^+)}}\\ 0\\ 0 \end{pmatrix},\quad \begin{pmatrix} 0\\ 0\\ \sqrt{\frac{\xi_{\boldsymbol{k}^-}-k_z^-}{2\xi_{\boldsymbol{k}^-}}}\\ -\frac{k_x^-+ik_y^-}{\sqrt{2\xi_{\boldsymbol{k}^-}(\xi_{\boldsymbol{k}^-}-k_z^-)}}\\ 0\\ 0\\ \frac{\Delta e^{-i(\phi+\chi)}}{\sqrt{2\xi_{\boldsymbol{k}^-}(\xi_{\boldsymbol{k}^-}-k_z^-)}}\\ 0 \end{pmatrix},\quad \begin{pmatrix} 0\\ 0\\ -\frac{k_x^--ik_y^-}{\sqrt{2\xi_{\boldsymbol{k}^-}(\xi_{\boldsymbol{k}^-}+k_z^-)}}\\ \sqrt{\frac{\xi_{\boldsymbol{k}^-}+k_z^-}{2\xi_{\boldsymbol{k}^-}}}\\ 0\\ 0\\ 0\\ \frac{\Delta e^{-i(\phi+\chi)}}{\sqrt{2\xi_{\boldsymbol{k}^-}(\xi_{\boldsymbol{k}^-}+k_z^-)}} \end{pmatrix},$$

while those for $-\varepsilon_{\boldsymbol{k}^\mp}$ are

$$\begin{pmatrix} 0\\ 0\\ -\sqrt{\frac{\xi_{\boldsymbol{k}^-}+k_z^-}{2\xi_{\boldsymbol{k}^-}}}\\ -\frac{k_x^-+ik_y^-}{\sqrt{2\xi_{\boldsymbol{k}^-}(\xi_{\boldsymbol{k}^-}+k_z^-)}}\\ 0\\ 0\\ \frac{\Delta e^{-i(\phi+\chi)}}{\sqrt{2\xi_{\boldsymbol{k}^-}(\xi_{\boldsymbol{k}^-}+k_z^-)}}\\ 0 \end{pmatrix},\quad \begin{pmatrix} 0\\ 0\\ -\frac{k_x^--ik_y^-}{\sqrt{2\xi_{\boldsymbol{k}^-}(\xi_{\boldsymbol{k}^-}-k_z^-)}}\\ -\sqrt{\frac{\xi_{\boldsymbol{k}^-}-k_z^-}{2\xi_{\boldsymbol{k}^-}}}\\ 0\\ 0\\ 0\\ \frac{\Delta e^{-i(\phi+\chi)}}{\sqrt{2\xi_{\boldsymbol{k}^-}(\xi_{\boldsymbol{k}^-}-k_z^-)}} \end{pmatrix},\quad \begin{pmatrix} -\sqrt{\frac{\xi_{\boldsymbol{k}^+}-k_z^+}{2\xi_{\boldsymbol{k}^+}}}\\ \frac{k_x^++ik_y^+}{\sqrt{2\xi_{\boldsymbol{k}^+}(\xi_{\boldsymbol{k}^+}-k_z^+)}}\\ 0\\ 0\\ \frac{\Delta e^{-i(\phi-\chi)}}{\sqrt{2\xi_{\boldsymbol{k}^+}(\xi_{\boldsymbol{k}^+}-k_z^+)}}\\ 0\\ 0\\ 0 \end{pmatrix},\quad \begin{pmatrix} \frac{k_x^+-ik_y^+}{\sqrt{2\xi_{\boldsymbol{k}^+}(\xi_{\boldsymbol{k}^+}+k_z^+)}}\\ -\sqrt{\frac{\xi_{\boldsymbol{k}^+}+k_z^+}{2\xi_{\boldsymbol{k}^+}}}\\ 0\\ 0\\ 0\\ \frac{\Delta e^{-i(\phi-\chi)}}{\sqrt{2\xi_{\boldsymbol{k}^+}(\xi_{\boldsymbol{k}^+}+k_z^+)}}\\ 0\\ 0 \end{pmatrix}.$$

So we construct the unitary matrix U that diagonalizes the unperturbed Dirac/Weyl-BdG Hamiltonian:

$$\hat{U} = \begin{pmatrix}
\sqrt{\frac{\xi_{\boldsymbol{k}^+}+k_z^+}{2\xi_{\boldsymbol{k}^+}}} & \frac{k_x^+-ik_y^+}{\sqrt{2\xi_{\boldsymbol{k}^+}(\xi_{\boldsymbol{k}^+}-k_z^+)}} & 0 & 0 & 0 & 0 & -\sqrt{\frac{\xi_{\boldsymbol{k}^+}-k_z^+}{2\xi_{\boldsymbol{k}^+}}} & \frac{k_x^+-ik_y^+}{\sqrt{2\xi_{\boldsymbol{k}^+}(\xi_{\boldsymbol{k}^+}+k_z^+)}} \\
\frac{k_x^++ik_y^+}{\sqrt{2\xi_{\boldsymbol{k}^+}(\xi_{\boldsymbol{k}^+}+k_z^+)}} & \sqrt{\frac{\xi_{\boldsymbol{k}^+}-k_z^+}{2\xi_{\boldsymbol{k}^+}}} & 0 & 0 & 0 & 0 & \frac{k_x^++ik_y^+}{\sqrt{2\xi_{\boldsymbol{k}^+}(\xi_{\boldsymbol{k}^+}-k_z^+)}} & -\sqrt{\frac{\xi_{\boldsymbol{k}^+}+k_z^+}{2\xi_{\boldsymbol{k}^+}}} \\
0 & 0 & \sqrt{\frac{\xi_{\boldsymbol{k}^-}-k_z^-}{2\xi_{\boldsymbol{k}^-}}} & \frac{-k_x^-+ik_y^-}{\sqrt{2\xi_{\boldsymbol{k}^-}(\xi_{\boldsymbol{k}^-}+k_z^-)}} & -\sqrt{\frac{\xi_{\boldsymbol{k}^-}+k_z^-}{2\xi_{\boldsymbol{k}^-}}} & \frac{-k_x^-+ik_y^-}{\sqrt{2\xi_{\boldsymbol{k}^-}(\xi_{\boldsymbol{k}^-}-k_z^-)}} & 0 & 0 \\
0 & 0 & -\frac{k_x^-+ik_y^-}{\sqrt{2\xi_{\boldsymbol{k}^-}(\xi_{\boldsymbol{k}^-}-k_z^-)}} & \sqrt{\frac{\xi_{\boldsymbol{k}^-}+k_z^-}{2\xi_{\boldsymbol{k}^-}}} & -\frac{k_x^-+ik_y^-}{\sqrt{2\xi_{\boldsymbol{k}^-}(\xi_{\boldsymbol{k}^-}+k_z^-)}} & -\sqrt{\frac{\xi_{\boldsymbol{k}^-}-k_z^-}{2\xi_{\boldsymbol{k}^-}}} & 0 & 0 \\
\frac{\Delta e^{-i(\phi-\chi)}}{\sqrt{2\xi_{\boldsymbol{k}^+}(\xi_{\boldsymbol{k}^+}+k_z^+)}} & 0 & 0 & 0 & 0 & 0 & \frac{\Delta e^{-i(\phi-\chi)}}{\sqrt{2\xi_{\boldsymbol{k}^+}(\xi_{\boldsymbol{k}^+}-k_z^+)}} & 0 \\
0 & \frac{\Delta e^{-i(\phi-\chi)}}{\sqrt{2\xi_{\boldsymbol{k}^+}(\xi_{\boldsymbol{k}^+}-k_z^+)}} & 0 & 0 & 0 & 0 & 0 & \frac{\Delta e^{-i(\phi-\chi)}}{\sqrt{2\xi_{\boldsymbol{k}^+}(\xi_{\boldsymbol{k}^+}+k_z^+)}} \\
0 & 0 & \frac{\Delta e^{-i(\phi+\chi)}}{\sqrt{2\xi_{\boldsymbol{k}^-}(\xi_{\boldsymbol{k}^-}-k_z^-)}} & 0 & \frac{\Delta e^{-i(\phi+\chi)}}{\sqrt{2\xi_{\boldsymbol{k}^-}(\xi_{\boldsymbol{k}^-}+k_z^-)}} & 0 & 0 & 0 \\
0 & 0 & 0 & \frac{\Delta e^{-i(\phi+\chi)}}{\sqrt{2\xi_{\boldsymbol{k}^-}(\xi_{\boldsymbol{k}^-}+k_z^-)}} & 0 & \frac{\Delta e^{-i(\phi+\chi)}}{\sqrt{2\xi_{\boldsymbol{k}^-}(\xi_{\boldsymbol{k}^-}-k_z^-)}} & 0 & 0
\end{pmatrix}$$

which $\xi_{\boldsymbol{k}^\pm} = \varepsilon_{\boldsymbol{k}^\pm} - \mu^\pm$. The matrix $\hat{U}$ satisfies $H\hat{U} = \hat{U}\hat{\varepsilon}$ with $\hat{\varepsilon} = \left[(\frac{\varepsilon_{\boldsymbol{k}^+}+\varepsilon_{\boldsymbol{k}^-}}{2})\tau_0 + (\frac{\varepsilon_{\boldsymbol{k}^+}-\varepsilon_{\boldsymbol{k}^-}}{2})\tau_z\right]\sigma_0\eta_z$.

Using $\hat{\boldsymbol{c}}_{\boldsymbol{k}} = \hat{U}\hat{\boldsymbol{\gamma}}_{\boldsymbol{k}}$ and substituting $\hat{U}$ in $\langle\hat{\boldsymbol{c}}_{\boldsymbol{k}}(t)\hat{\boldsymbol{c}}^\dagger_{\boldsymbol{k}}(t')\rangle$ expression, we can express the correlation functions in (S20) as, $\langle\hat{\boldsymbol{c}}_{\boldsymbol{k}}(t)\hat{\boldsymbol{c}}^\dagger_{\boldsymbol{k}}(t')\rangle = \hat{U}_{\boldsymbol{k}}\langle\hat{\boldsymbol{\gamma}}_{\boldsymbol{k}}(t)\hat{\boldsymbol{\gamma}}^\dagger_{\boldsymbol{k}}(t')\rangle\hat{U}^\dagger_{\boldsymbol{k}}$ and $\langle\hat{\boldsymbol{c}}^*_{\boldsymbol{k}}(t)\hat{\boldsymbol{c}}^T_{\boldsymbol{k}}(t')\rangle^T = [\hat{U}^*_{\boldsymbol{k}}\langle\hat{\boldsymbol{\gamma}}^*_{\boldsymbol{k}}(t)\hat{\boldsymbol{\gamma}}^T_{\boldsymbol{k}}(t')\rangle\hat{U}^T_{\boldsymbol{k}}]^T = \hat{U}_{\boldsymbol{k}}\langle\hat{\boldsymbol{\gamma}}^*_{\boldsymbol{k}}(t)\hat{\boldsymbol{\gamma}}^T_{\boldsymbol{k}}(t')\rangle^T\hat{U}^\dagger_{\boldsymbol{k}}$ where $\hat{\boldsymbol{\gamma}}_{\boldsymbol{k}}(t) = (\check{\boldsymbol{\gamma}}_{\boldsymbol{k}^+}e^{-i\xi_{\boldsymbol{k}^+}t/\hbar}, \check{\boldsymbol{\gamma}}_{\boldsymbol{k}^-}e^{-i\xi_{\boldsymbol{k}^-}t/\hbar}, \check{\boldsymbol{\gamma}}^\dagger_{\boldsymbol{k}^+}e^{i\xi_{\boldsymbol{k}^+}t/\hbar}, \check{\boldsymbol{\gamma}}^\dagger_{\boldsymbol{k}^-}e^{i\xi_{\boldsymbol{k}^-}t/\hbar})^T$. It should be noted that $\check{\boldsymbol{\gamma}}$ represents a two-component spinor while $\hat{\boldsymbol{\gamma}}$ holds for a eight-component spinor and $\gamma_{\boldsymbol{k}^\pm,\alpha,\lambda}$s are the components of $\check{\boldsymbol{\gamma}}$. Given that $\langle\boldsymbol{\gamma}_{\boldsymbol{k}^\pm,\alpha,\lambda}\boldsymbol{\gamma}^\dagger_{\boldsymbol{k}^\pm,\alpha',\lambda'}\rangle = n(-\xi_{\boldsymbol{k}^\pm})\delta_{\alpha\alpha'}\delta_{\lambda\lambda'}$, $\langle\boldsymbol{\gamma}^\dagger_{\boldsymbol{k}^\pm,\alpha,\lambda}\boldsymbol{\gamma}_{\boldsymbol{k}^\pm,\alpha',\lambda'}\rangle = n(\xi_{\boldsymbol{k}^\pm})\delta_{\alpha\alpha'}\delta_{\lambda\lambda'}$ and $\langle\boldsymbol{\gamma}_{\boldsymbol{k}^\pm,\alpha,\lambda}\boldsymbol{\gamma}_{\boldsymbol{k}^\pm,\alpha',\lambda'}\rangle = \langle\boldsymbol{\gamma}^\dagger_{\boldsymbol{k}^\pm,\alpha,\lambda}\boldsymbol{\gamma}^\dagger_{\boldsymbol{k}^\pm,\alpha',\lambda'}\rangle = 0$, one can see that $\langle\hat{\boldsymbol{\gamma}}^*_{\boldsymbol{k}}(t)\hat{\boldsymbol{\gamma}}^T_{\boldsymbol{k}}(t')\rangle$ is a 8×8 diagonal matrix in which the first two entries of the main diagonal are $n(-\xi_{\boldsymbol{k}^+})e^{-i\xi_{\boldsymbol{k}^+}(t-t')}$, the next two are $n(-\xi_{\boldsymbol{k}^-})e^{-i\xi_{\boldsymbol{k}^-}(t-t')}$, the next two are $n(\xi_{\boldsymbol{k}^-})e^{i\xi_{\boldsymbol{k}^-}(t-t')}$ and the last two entries are $n(\xi_{\boldsymbol{k}^+})e^{i\xi_{\boldsymbol{k}^+}(t-t')}$. Ultimately, we obtain

$$\langle\hat{\boldsymbol{c}}_{\boldsymbol{k}}(t)\hat{\boldsymbol{c}}^\dagger_{\boldsymbol{k}}(t')\rangle = \begin{bmatrix} g_1(\varepsilon_{\boldsymbol{k}}, t-t') & f(\varepsilon_{\boldsymbol{k}}, t-t') \\ f^\dagger(\varepsilon_{\boldsymbol{k}}, -t+t') & g_2(\varepsilon_{\boldsymbol{k}}, t-t') \end{bmatrix}, \tag{S25}$$

where,

$$g_1(\varepsilon_{\boldsymbol{k}}, t) = [1 + \frac{\Delta_l^2}{2(\varepsilon_{\boldsymbol{k}}^2 - k_z^2)}]n_+(\varepsilon_{\boldsymbol{k}}, t)\sigma_0\tau_0 - \frac{n_-(\varepsilon_{\boldsymbol{k}}, t)}{\varepsilon_{\boldsymbol{k}}}(\boldsymbol{k}\cdot\boldsymbol{\sigma})\tau_z - \frac{\Delta_l^2 k_z n_-(\varepsilon_{\boldsymbol{k}}, t)}{2\varepsilon_{\boldsymbol{k}}(\varepsilon_{\boldsymbol{k}}^2 - k_z^2)}\sigma_z\tau_z$$
$$g_2(\varepsilon_{\boldsymbol{k}}, t) = \frac{\Delta_l^2}{2(\varepsilon_{\boldsymbol{k}}^2 - k_z^2)}\left[n_+(\varepsilon_{\boldsymbol{k}}, t)\sigma_0\tau_0 - \frac{k_z n_-(\varepsilon_{\boldsymbol{k}}, t)}{\varepsilon_{\boldsymbol{k}}}\sigma_z\tau_z\right] \tag{S26}$$

while $f(\xi_{\boldsymbol{k}}, t)$ is a diagonal matrix and its diagonal entries are,

$$f_{11}(\xi_{\boldsymbol{k}^+}, t) = \frac{1}{2}\left[-\frac{n_-(\xi_{\boldsymbol{k}^+}, t)\sigma_0}{\xi_{\boldsymbol{k}^+}} - \frac{n_-(\xi_{\boldsymbol{k}^+}, t)ik_z^+(k_x^+\sigma_y - k_y^+\sigma_x)}{\xi_{\boldsymbol{k}^+}(\xi_{\boldsymbol{k}^+}^2 - {k_z^+}^2)} + \frac{n_+(\xi_{\boldsymbol{k}^+}, t)(k_x^+\sigma_x + k_y^+\sigma_y)}{(\xi_{\boldsymbol{k}^+}^2 - {k_z^+}^2)}\right]\Delta_l e^{i(\phi-\chi)} \tag{S27}$$
$$f_{22}(\varepsilon_{\boldsymbol{k}^-}, t) = \frac{1}{2}\left[-\frac{n_-(\xi_{\boldsymbol{k}^-}, t)\sigma_0}{\xi_{\boldsymbol{k}^-}} - \frac{n_-(\xi_{\boldsymbol{k}^-}, t)ik_z^-(k_x^-\sigma_y - k_y^-\sigma_x)}{\xi_{\boldsymbol{k}^-}(\xi_{\boldsymbol{k}^-}^2 - {k_z^-}^2)} + \frac{n_+(\xi_{\boldsymbol{k}^-}, t)(k_x^-\sigma_x + k_y^-\sigma_y)}{(\xi_{\boldsymbol{k}^-}^2 - {k_z^-}^2)}\right]\Delta_l e^{i(\phi+\chi)}$$

with,

$$n_\pm(\xi_{\boldsymbol{k}}, t) = n(\xi_{\boldsymbol{k}})e^{i\xi_{\boldsymbol{k}}t/\hbar} \pm n(-\xi_{\boldsymbol{k}})e^{-i\xi_{\boldsymbol{k}}t/\hbar}. \tag{S28}$$

Finally the correlator $\langle\hat{\boldsymbol{d}}^*_{\boldsymbol{q}}(t)\hat{\boldsymbol{d}}^T_{\boldsymbol{q}}(t')\rangle^T$ can be obtained by replacements $n_-(\xi_{\boldsymbol{k}^\pm}) \to -n_-(\xi_{\boldsymbol{q}^\pm})$ and $\boldsymbol{k} \to \boldsymbol{q}$ in the above equations. Hence,

$$\langle\hat{\boldsymbol{d}}^*_{\boldsymbol{q}}(t)\hat{\boldsymbol{d}}^T_{\boldsymbol{q}}(t')\rangle^T = \begin{bmatrix} {g'}_1(\varepsilon_{\boldsymbol{q}}, t-t') & f'(\varepsilon_{\boldsymbol{q}}, t-t') \\ {f'}^\dagger(\varepsilon_{\boldsymbol{q}}, -t+t') & {g'}_2(\varepsilon_{\boldsymbol{q}}, t-t') \end{bmatrix}, \tag{S29}$$

where f' and $g'_{1/2}$ are the same as$f$ and $g_{1/2}$ in S25 but for $\xi_{\boldsymbol{q}}$ and with the exception that $n_-(\xi_{\boldsymbol{q}^\pm})$ is changed to $-n_-(\xi_{\boldsymbol{q}^\pm})$. By Substituting in (S20), the Tr term is obtained as,

$$\begin{aligned}\mathrm{Tr}\Big[\langle\hat{\boldsymbol{c}}_{\boldsymbol{k}}(t)\hat{\boldsymbol{c}}^\dagger_{\boldsymbol{k}}(t')\rangle\hat{T}_{\boldsymbol{kq}}\sigma_z\langle\hat{\boldsymbol{d}}^*_{\boldsymbol{q}}(t)\hat{\boldsymbol{d}}^T_{\boldsymbol{q}}(t')\rangle^T\hat{T}_{\boldsymbol{qk}}-\langle\hat{\boldsymbol{c}}^*_{\boldsymbol{k}}(t')\hat{\boldsymbol{c}}^T_{\boldsymbol{k}}(t)\rangle^T\hat{T}_{\boldsymbol{kq}}\sigma_z\langle\hat{\boldsymbol{d}}_{\boldsymbol{q}}(t')\hat{\boldsymbol{d}}^\dagger_{\boldsymbol{q}}(t)\rangle\hat{T}_{\boldsymbol{qk}}\Big] &= \\ e^{ieV(t-t')/\hbar}[g_1(\xi_{\boldsymbol{k}},t-t')g'_1(\xi_{\boldsymbol{q}},t-t')-g'_1(\xi_{\boldsymbol{k}},t'-t)g_1(\xi_{\boldsymbol{q}},t'-t)] \\ -e^{-ieV(t-t')/\hbar}[g_2(\xi_{\boldsymbol{k}},t-t')g'_2(\xi_{\boldsymbol{q}},t-t')-g'_2(\xi_{\boldsymbol{k}},t'-t)g_2(\xi_{\boldsymbol{q}},t'-t)] \\ -e^{ieV(t+t')/\hbar}[f(\xi_{\boldsymbol{k}},t-t')f'^\dagger(\xi_{\boldsymbol{q}},-t+t')-f'(\xi_{\boldsymbol{k}},t'-t)f^\dagger(\xi_{\boldsymbol{q}},-t'+t)] \\ +e^{-ieV(t+t')/\hbar}[f^\dagger(\xi_{\boldsymbol{k}},-t+t')f'(\xi_{\boldsymbol{q}},t-t')-f'^\dagger(\xi_{\boldsymbol{k}},-t'+t)f(\xi_{\boldsymbol{q}},t'-t)].\end{aligned} \tag{S30}$$

The first two lines get the single electron current that is not our purpose. Indeed, we are looking for Josephson current which is a supercurrent. Hence, we focus on the last two lines. Furthermore, to obtain the Josephson current as (S20) shows, we have summations over all components of $\boldsymbol{k}$ and $\boldsymbol{q}$ from $-\infty$ to $+\infty$. We should mention that we work in continuum limit. These summations can be changed to summations on $\boldsymbol{k}^\pm_{x,y,z}$ and $\boldsymbol{q}^\pm_{x,y,z}$ from $-\infty$ to $+\infty$ with a change in variables from $(\boldsymbol{k}^\pm,\boldsymbol{q}^\pm)\to(\boldsymbol{k}',\boldsymbol{q}')$ but the limits of summations remain $-\infty$ to $+\infty$. By this explanation, we omit the terms with odd order of $k^\pm_{x,y,z}$ and $q^\pm_{x,y,z}$. They give null contributions to summations on $\boldsymbol{k}^\pm$ and $\boldsymbol{q}^\pm$. With these considerations and making a change of variables with $t-t'=t_1$ the current becomes,:

$$\begin{aligned}I_J=\frac{e}{\hbar^2}\frac{|t|^2}{V_lV_r}\int_0^\infty dt_1e^{-0+t_1}\ \Big\{\ & e^{ieV(2t-t_1)/\hbar}\sum_{\boldsymbol{k}^+,\boldsymbol{q}^+}[\tilde{f}(\xi_{\boldsymbol{k}^+},t_1)-\tilde{f}(\xi_{\boldsymbol{k}^+},-t_1)+\tilde{f}(\xi_{\boldsymbol{k}^-},t_1)-\tilde{f}(\xi_{\boldsymbol{k}^-},-t_1)] \\ &+\ e^{-ieV(2t-t_1)/\hbar}\sum_{\boldsymbol{k}^-,\boldsymbol{q}^-}[\tilde{f}^*(\xi_{\boldsymbol{k}^+},t_1)-\tilde{f}^*(\xi_{\boldsymbol{k}^+},-t_1)+\tilde{f}^*(\xi_{\boldsymbol{k}^-},t_1)-\tilde{f}^*(\xi_{\boldsymbol{k}^-},-t_1)]\Big\}\end{aligned} \tag{S31}$$

$$\begin{aligned}=\frac{e}{\hbar^2}\frac{|t|^2}{V_lV_r}\mathrm{Re}\Big\{\int_0^\infty dt_1\qquad & e^{-0+t_1}e^{ieV(2t-t_1)/\hbar} \\ &\times\ \Big\{\sum_{\boldsymbol{k}^+,\boldsymbol{q}^+}[\tilde{f}(\xi_{\boldsymbol{k}^+},t_1)-\tilde{f}(\xi_{\boldsymbol{k}^+},-t_1)]+\sum_{\boldsymbol{k}^-,\boldsymbol{q}^-}[\tilde{f}(\xi_{\boldsymbol{k}^-},t_1)-\tilde{f}(\xi_{\boldsymbol{k}^-},-t_1)]\Big\},\end{aligned} \tag{S32}$$

with,

$$\begin{aligned}[\tilde{f}(\xi_{\boldsymbol{k}^\pm},t_1)-\tilde{f}(\xi_{\boldsymbol{k}^\pm},-t_1)] &= \frac{\Delta_l\Delta_r}{2\xi_{\boldsymbol{k}^\pm}\xi_{\boldsymbol{q}^\pm}}e^{i[(\phi_l-\phi_r)\mp(\chi_l-\chi_r)]} \\ \times\ \Big\{[1-n(\xi_{\boldsymbol{k}^\pm})-n(\xi_{\boldsymbol{q}^\pm})][e^{-i(\xi_{\boldsymbol{k}^\pm}+\xi_{\boldsymbol{q}^\pm})t_1/\hbar}-e^{i(\xi_{\boldsymbol{k}^\pm}+\xi_{\boldsymbol{q}^\pm})t_1/\hbar}] &+[n(\xi_{\boldsymbol{k}^\pm})-n(\xi_{\boldsymbol{q}^\pm})][e^{-i(\xi_{\boldsymbol{k}^\pm}-\xi_{\boldsymbol{q}^\pm})t_1/\hbar}-e^{i(\xi_{\boldsymbol{k}^\pm}-\xi_{\boldsymbol{q}^\pm})t_1/\hbar}]\Big\}.\end{aligned} \tag{S33}$$

We safely set $e^{0+t}\to1$ in the integral. To integrate on t_1, we use $\int_0^\infty dt_1e^{-i(x-i0_+)t_1}=-i/x$. The integral is thereby given by,

$$\begin{aligned}I_J\ =\ & \frac{e}{\hbar^2}\frac{|t|^2}{V_lV_r}\Delta_l\Delta_r\mathrm{Re}\Big\{e^{i(\phi_l-\phi_r+2eVt/\hbar)} \\ &\times\ \Big\{\sum_{\boldsymbol{k}^+,\boldsymbol{q}^+}\frac{e^{i(\chi_l-\chi_r)}}{\xi_{\boldsymbol{k}^+}\xi_{\boldsymbol{q}^+}}\{[1-n(\xi_{\boldsymbol{k}^+})-n(\xi_{\boldsymbol{q}^+})][\frac{-i\hbar}{eV+(\xi_{\boldsymbol{k}^+}+\xi_{\boldsymbol{q}^+})}-\frac{-i\hbar}{eV-(\xi_{\boldsymbol{k}^+}+\xi_{\boldsymbol{q}^+})}] \\ &+\ [n(\xi_{\boldsymbol{k}^+})-n(\xi_{\boldsymbol{q}^+})][\frac{-i\hbar}{eV+(\xi_{\boldsymbol{k}^+}-\xi_{\boldsymbol{q}^+})}-\frac{-i\hbar}{eV-(\xi_{\boldsymbol{k}^+}-\xi_{\boldsymbol{q}^+})}]\} \\ &+\ \sum_{\boldsymbol{k}^-,\boldsymbol{q}^-}\frac{e^{-i(\chi_l-\chi_r)}}{\xi_{\boldsymbol{k}}\xi_{\boldsymbol{q}^-}}\{[1-n(\xi_{\boldsymbol{k}^-})-n(\xi_{\boldsymbol{q}^-})][\frac{-i\hbar}{eV+(\xi_{\boldsymbol{k}^-}+\xi_{\boldsymbol{q}^-})}-\frac{-i\hbar}{eV-(\xi_{\boldsymbol{k}^-}+\xi_{\boldsymbol{q}^-})}] \\ &+\ [n(\xi_{\boldsymbol{k}^-})-n(\xi_{\boldsymbol{q}^-})][\frac{-i\hbar}{eV+(\xi_{\boldsymbol{k}^-}-\xi_{\boldsymbol{q}^-})}-\frac{-i\hbar}{eV-(\xi_{\boldsymbol{k}^-}-\xi_{\boldsymbol{q}^-})}]\}\Big\}\Big\}.\end{aligned} \tag{S34}$$

Sum over $\boldsymbol{k}^\pm$ and $\boldsymbol{q}^\pm$ are turned into integration over the corresponding energy variables $\epsilon_{\boldsymbol{k}^\pm}=\hbar\boldsymbol{k}^\pm$ and $\epsilon_{\boldsymbol{q}^\pm}=\hbar\boldsymbol{q}^\pm$ as $(1/V_lV_r)\sum_{\boldsymbol{k}^\pm\boldsymbol{q}^\pm}\to\int_{-\infty}^{+\infty}d\epsilon_{\boldsymbol{k}^\pm}N(\epsilon_{\boldsymbol{k}^\pm})\int_{-\infty}^{+\infty}d\epsilon_{\boldsymbol{q}^\pm}N(\epsilon_{\boldsymbol{q}^\pm})$, where the density of states per unit volume, spin and chirality

component is $N(\epsilon_{\boldsymbol{k}^\pm/\boldsymbol{q}^\pm}) = (1/V_{l/r})\sum_{\boldsymbol{k}^\pm/\boldsymbol{q}^\pm}\delta(\epsilon_{\boldsymbol{k}^\pm/\boldsymbol{q}^\pm})$. Then we change the variables $\epsilon_{\boldsymbol{k}^\pm}$ and $\epsilon_{\boldsymbol{q}^\pm}$ to $\xi_{\boldsymbol{k}^\pm}$ and $\xi_{\boldsymbol{q}^\pm}$ as, $d\epsilon_{\boldsymbol{k}^\pm/\boldsymbol{q}^\pm} = d\xi_{\boldsymbol{k}^\pm/\boldsymbol{q}^\pm}\frac{d\epsilon_{\boldsymbol{k}^\pm/\boldsymbol{q}^\pm}}{d\xi_{\boldsymbol{k}^\pm/\boldsymbol{q}^\pm}} = d\xi_{\boldsymbol{k}^\pm/\boldsymbol{q}^\pm}\Theta(\xi_{\boldsymbol{k}^\pm/\boldsymbol{q}^\pm} - \Delta_{l/r})\frac{\xi_{\boldsymbol{k}^\pm/\boldsymbol{q}^\pm}}{\sqrt{\xi^2_{\boldsymbol{k}^\pm/\boldsymbol{q}^\pm} - \Delta^2_{l/r}}}$. Up to here, it seems that in I_J in Eq. (S34) we deal with four different integrals for integration on positive indices and four different integrals in integration on negative indices, but we will show that these four integrals for each type of indices are the same. Despite from the indices these four integrals are as follows:

$$\int_{-\infty}^{+\infty} d\xi_{\boldsymbol{k}}\frac{\Theta(\xi_{\boldsymbol{k}} - \Delta_r)}{\sqrt{\xi^2_{\boldsymbol{k}} - \Delta^2_l}}N(\epsilon_{\boldsymbol{k}})\int_{-\infty}^{+\infty} d\xi_{\boldsymbol{q}}\frac{\Theta(\xi_{\boldsymbol{q}} - \Delta_r)}{\sqrt{\xi^2_{\boldsymbol{q}} - \Delta^2_r}}N(\epsilon_{\boldsymbol{q}}) \tag{S35}$$
$$\times\left\{[[1 - n(\xi_{\boldsymbol{k}}) - n(\xi_{\boldsymbol{q}})][\frac{-i\hbar}{eV + (\xi_{\boldsymbol{k}} + \xi_{\boldsymbol{q}})} - \frac{-i\hbar}{eV - (\xi_{\boldsymbol{k}} + \xi_{\boldsymbol{q}})}] + [n(\xi_{\boldsymbol{k}}) - n(\xi_{\boldsymbol{q}})][\frac{-i\hbar}{eV + (\xi_{\boldsymbol{k}} - \xi_{\boldsymbol{q}})} - \frac{-i\hbar}{eV - (\xi_{\boldsymbol{k}} - \xi_{\boldsymbol{q}})}]]\right\}$$

At the first step we can replace $1 - n(\xi_{\boldsymbol{k}})$ in the first term, with $n(-\xi_{\boldsymbol{k}})$ and then change variable $-\xi_{\boldsymbol{k}} \to \xi$ and $\xi_{\boldsymbol{q}} \to \xi'$. Since the integration is over $\xi_{\boldsymbol{k}}$ and $\xi_{\boldsymbol{q}}$, the variable change $-\xi_{\boldsymbol{k}} \to \xi$ produces a negative sign. Then we have, $[n(\xi') - n(\xi)][1/(eV - \xi + \xi') - 1/(eV + \xi - \xi')]$ which is the same as the second two terms in (S35). On the other hand the first term in $[n(\xi') - n(\xi)][1/(eV - \xi + \xi') - 1/(eV + \xi - \xi')]$ is transformed to the second one by changing variable $\xi \to \xi'$. Hence, we are only left with two double-fold integrals that carries a multiplicative factor of 4:

$$\begin{aligned} I_J = &\frac{4e|t|^2}{\hbar}\Delta_l\Delta_r\left[\frac{e^{i(\chi_l-\chi_r)} + e^{-i(\chi_l-\chi_r)}}{2}\right]\int_{-\infty}^{+\infty} d\varepsilon\frac{\Theta(\varepsilon - \Delta_l)}{\sqrt{\varepsilon^2 - \Delta^2_l}}N(\xi_F)\int_{-\infty}^{+\infty} d\varepsilon\frac{\Theta(\varepsilon - \Delta_r)}{\sqrt{\varepsilon^2 - \Delta^2_r}}N(\xi_F) \\ &\times\mathrm{Re}\left\{-ie^{i(\phi_l-\phi_r+2eVt/\hbar)}\frac{n(\varepsilon') - n(\varepsilon)}{eV + \varepsilon - \varepsilon'}\right\} \\ = &\frac{4e|t|^2}{\hbar}\Delta_l\Delta_r\cos(\chi_l - \chi_r)\int_{-\infty}^{+\infty} d\varepsilon\frac{\Theta(\varepsilon - \Delta_l)}{\sqrt{\varepsilon^2 - \Delta^2_l}}N(\xi_F)\int_{-\infty}^{+\infty} d\varepsilon\frac{\Theta(\varepsilon - \Delta_r)}{\sqrt{\varepsilon^2 - \Delta^2_r}}N(\xi_F) \\ &\times\mathrm{Re}\left\{-ie^{i(\phi_l-\phi_r+2eVt/\hbar)}\frac{n(\varepsilon') - n(\varepsilon)}{eV + \varepsilon - \varepsilon'}\right\}.. \end{aligned} \tag{S36}$$

In order to evaluate the above integral, for simplicity we replace $N(\xi_{\boldsymbol{k}})$ and $N(\xi_{\boldsymbol{q}})$ with the density of states at the Fermi energy $N(\xi_F)$. Let us define $\int_{-\infty}^{+\infty} d\varepsilon\mathcal{F}_{l/r}(\varepsilon)$ as a shorthand for $\int_{-\infty}^{+\infty} d\varepsilon\Theta(\varepsilon - \Delta_{l/r})\frac{\Delta_{l/r}}{\sqrt{\varepsilon^2-\Delta^2_{l/r}}}N(\xi_F)$. Then replacing and $\frac{1}{eV\pm(\varepsilon_{\boldsymbol{k}}\pm\varepsilon_{\boldsymbol{q}})}$ with $P\frac{1}{eV\pm(\varepsilon_{\boldsymbol{k}}\pm\varepsilon_{\boldsymbol{q}})} + i\pi\delta[eV \pm (\varepsilon_{\boldsymbol{k}} \pm \varepsilon_{\boldsymbol{q}})]$ where P denoting the principal value, the current becomes,

$$\begin{aligned} I_J &= \frac{4e|t|^2}{\hbar}\Delta_l\Delta_r\cos(\chi_l - \chi_r)\int_{-\infty}^{+\infty} d\varepsilon\mathcal{F}_l(\varepsilon)\int_{-\infty}^{+\infty} d\varepsilon\mathcal{F}_r(\varepsilon') \\ &\times\left\{P\frac{n(\varepsilon') - n(\varepsilon)}{eV + \varepsilon - \varepsilon'}\sin[\phi_l - \phi_r + \frac{2eVt}{\hbar}]\right. \\ &+ \left.\pi[n(\varepsilon') - n(\varepsilon)]\delta(eV + \varepsilon - \varepsilon')\cos[\phi_l - \phi_r + \frac{2eVt}{\hbar}]\right\}. \end{aligned} \tag{S37}$$

As the supercurrent splits to two distinct parts, one part proportional to $\sin[\phi_l - \phi_r + 2eVt/\hbar]$ and the other one proportional to $\cos[\phi_l - \phi_r + 2eVt/\hbar]$, it is appropriate to rewrite the supercurrent equation as,

$$I_J = I_s\cos(\chi_l - \chi_r), \tag{S38}$$

where I_s is the usual supercurrent ($\Delta\phi = \phi_l - \phi_r$),

$$I_s = I_{ss}\sin[\Delta\phi + \frac{2eVt}{\hbar}] + I_{sc}\cos[\Delta\phi + \frac{2eVt}{\hbar}], \tag{S39}$$

with the coefficients

$$I_{ss} = \frac{4e|t|^2}{\hbar}P\int_{-\infty}^{+\infty} d\varepsilon\mathcal{F}_l(\varepsilon)\int_{-\infty}^{+\infty} d\varepsilon'\mathcal{F}_r(\varepsilon')\ \frac{n(\varepsilon') - n(\varepsilon)}{eV + \varepsilon - \varepsilon'}, \tag{S40}$$

$$I_{sc} = \frac{4\pi e|t|^2}{\hbar}\int_{-\infty}^{+\infty} d\varepsilon\ \mathcal{F}_l(\varepsilon)\mathcal{F}_l(\varepsilon + eV)[n(\varepsilon + eV) - n(\varepsilon)]. \tag{S41}$$

It is clear that in zero bias ($V = 0$), the coefficient of the cosine term vanishes but the sine term remains finite. The result (S38) is remarkable in the sense that it extends the usual supercurrent I_s of Eq. (S39) by a $\cos(\chi_l - \chi_r)$ term.

COMPUTATION OF THE CHIRAL SUPERCURRENT

So far we have used the tunneling and perturbation theory to evaluate the standard Josephson supercurrent. The same machinery with a slight change can be used to derive the chiral supercurrent. We start again from (S15) and (S16) keeping in mind that the chiral supercurrent is caused by the difference between positive- and negative-chirality supercurrents. Hence, we require to add a negative sign before positive chirality components of the supercurrent (S17). The matrix $\tau_z\eta_z$ does this job. Because of the special form of the Nambu basis function $\Psi = (\Psi_e, \Psi_h)$ we adopted, as noted above Eq. (2) in the main text, $\tau_z\eta_z$ would multiply a minus sign in front of the terms which have negative chirality. By this sign changing the chiral supercurrent is then obtained as,

$$I_5 = \frac{e}{\hbar^2}\frac{|t|^2}{V_l V_r} \sum_{\boldsymbol{k},\boldsymbol{q}} \int_{-\infty}^{t} dt' e^{0+t'} \tag{S42}$$
$$\times \ \mathrm{Tr}\left[\hat{\Gamma}(t)\langle\hat{\boldsymbol{c}}_{\boldsymbol{k}}(t)\hat{\boldsymbol{c}}^{\dagger}_{\boldsymbol{k}}(t')\rangle\hat{\Gamma}^{*}(t')(\tau_z\eta_z)\eta_z\langle\hat{\boldsymbol{d}}^{*}_{\boldsymbol{q}}(t)\hat{\boldsymbol{d}}^{T}_{\boldsymbol{q}}(t')\rangle^{T} - \hat{\Gamma}(t)\hat{\boldsymbol{c}}^{*}_{\boldsymbol{k}}(t')\hat{\boldsymbol{c}}^{T}_{\boldsymbol{k}}(t)\rangle^{T}\hat{\Gamma}^{*}(t')(\tau_z\eta_z)\eta_z\langle\hat{\boldsymbol{d}}_{\boldsymbol{q}}(t')\hat{\boldsymbol{d}}^{\dagger}_{\boldsymbol{q}}(t)\rangle\right].$$

Then we instead of (S31), we should compute,

$$\mathrm{Tr}\left[\hat{\Gamma}(t)\langle\hat{\boldsymbol{c}}_{\boldsymbol{k}}(t)\hat{\boldsymbol{c}}^{\dagger}_{\boldsymbol{k}}(t')\rangle\hat{\Gamma}^{*}(t')(\tau_z\eta_z)\eta_z\langle\hat{\boldsymbol{d}}^{*}_{\boldsymbol{q}}(t)\hat{\boldsymbol{d}}^{T}_{\boldsymbol{q}}(t')\rangle^{T}\right].$$

There are some differences between this equation and Eq. (S31), that leads to a characteristic and nice distinction between the chiral current and the total current. To reveal these fine differences, since we are eventually interested in two-particle currents, we focus on the $\tilde{f}$ part of (S31) and explain how it changes upon insertion of a $\tau_z\eta_z$ matrix within the brackets $\langle\hat{\boldsymbol{c}}_{\boldsymbol{k}}(t)\hat{\boldsymbol{c}}^{\dagger}_{\boldsymbol{k}}(t')\rangle$ and $\langle\hat{\boldsymbol{d}}^{*}_{\boldsymbol{q}}(t)\hat{\boldsymbol{d}}^{T}_{\boldsymbol{q}}(t')\rangle^{T}$. The expression $\hat{\Gamma}(t)\langle\hat{\boldsymbol{c}}_{\boldsymbol{k}}(t)\hat{\boldsymbol{c}}^{\dagger}_{\boldsymbol{k}}(t')\rangle\hat{\Gamma}^{*}(t')$ has the following matrix form in Nambu space,

$$\begin{pmatrix} e^{ieV(t-t')/\hbar}g_1(\varepsilon_{\boldsymbol{k}}) & e^{ieV(t+t')/\hbar}f(\varepsilon_{\boldsymbol{k}}) \\ e^{-ieV(t+t')/\hbar}f^{*}(\varepsilon_{\boldsymbol{k}}) & e^{-ieV(t-t')/\hbar}g_2(\varepsilon_{\boldsymbol{k}}) \end{pmatrix}, \tag{S43}$$

while $\langle\hat{\boldsymbol{d}}^{*}_{\boldsymbol{q}}(t)\hat{\boldsymbol{d}}^{T}_{\boldsymbol{q}}(t')\rangle^{T}$ is,

$$\begin{pmatrix} g_1'(\varepsilon_{\boldsymbol{q}}) & f'(\varepsilon_{\boldsymbol{q}}) \\ f^{*\prime}(\varepsilon_{\boldsymbol{q}}) & g_2'(\varepsilon_{\boldsymbol{q}}) \end{pmatrix}, \tag{S44}$$

where g_1' is the same as g_1 except that $n_- \to -n_-$ and $i \to -i$. If we have a η_z between matrices (S43) and (S44) and follow the steps in (S31), it causes a negative sign for $f^{*\prime}(\varepsilon_{\boldsymbol{q}})$, so instead of what we have in Eq. (S31), in the first step we would obtain $-e^{ieV(t+t')/\hbar}f(\varepsilon_{\boldsymbol{k}})\eta_z f^{*\prime}(\varepsilon_{\boldsymbol{q}}) + e^{-ieV(t+t')/\hbar}f^{*}(\varepsilon_{\boldsymbol{k}})\eta_z f'(\varepsilon_{\boldsymbol{q}})$. In the second step, one needs to insert $\tau_z\eta_z$. But since the current itself already has an $\hat{\eta}_z$ matrix, multiplication with an additional $\hat{\tau}_z\eta_z$, gives $\tau_z\eta_0$. Hence the negative sign in the first term is cancelled out and a τ_z is left behind, giving finally $e^{ieV(t+t')/\hbar}f(\varepsilon_{\boldsymbol{k}})\tau_z f^{*\prime}(\varepsilon_{\boldsymbol{q}}) + e^{-ieV(t+t')/\hbar}f^{*}(\varepsilon_{\boldsymbol{k}})\tau_z f'(\varepsilon_{\boldsymbol{q}})$. Since just the first term of f and f^{*} functions produce nonzero terms (because they are even in k_x and k_y), we are left with

$$\mathrm{Tr}\left[-\frac{n_-(\varepsilon_{\boldsymbol{k}})n_-(\varepsilon_{\boldsymbol{q}})}{4\varepsilon_{\boldsymbol{k}}\varepsilon_{\boldsymbol{q}}}\Delta_l\Delta_r e^{i(\phi_l+\chi_l\tau_z)}\tau_z e^{-i(\phi_r+\chi_r\tau_z)}\right],$$

where

$$\begin{aligned} &\mathrm{Tr}\left[e^{i\chi_l\tau_z}\tau_z e^{-i\chi_r\tau_z}\right] \\ &= \mathrm{Tr}[(\cos\chi_l + i\sin\chi_l\tau_z)(\cos\chi_r\tau_z - i\sin\chi_r)] \\ &= i\sin(\chi_l - \chi_r). \end{aligned} \tag{S45}$$

This should be contrasted with the case of Eq. (S31) where we had $\cos(\chi_l - \chi_r)$. The rest of calculations is the same as the derivation of I_J and the chiral supercurrent obtains as,

$$I_5 = I_s\sin(\chi_l - \chi_r). \tag{S46}$$

It is nice to notice that the forms of Eq. (S38) and (S46) suggest regard them as real and imaginary parts of another entity of the form

$$I_{\mathrm{complex}} = I_s e^{i(\chi_l-\chi_r)}, \tag{S47}$$

thereby the standard/chiral Josephson current becomes the real/imaginary part of (S47). This means that the standard and chiral Josephson currents are locked to each other by a phase lag of $\pi/2$ in the chiral angle difference $\chi_l - \chi_r$.

FIERZ TABLE

The general form of a Lorentz covariant lagrangian describing the pairing between two Dirac spinor (four fermion interaction) contains scalar, pseudo-scalar, vector, axial-vector and tensor structures under Lorentz transformations for the bilinear couplings:

$$\begin{aligned}\mathcal{L}_{int} &= g_s(\bar{\psi}_1\psi_2)(\bar{\psi}_3\psi_4) + g_5(\bar{\psi}_1\gamma_5\psi_2)(\bar{\psi}_3\gamma_5\psi_4) + g_\mu(\bar{\psi}_1\gamma^\mu\psi_2)(\bar{\psi}_3\gamma^\mu\psi_4) \\ &+ g_{5\mu}(\bar{\psi}_1\gamma_5\gamma^\mu\psi_2)(\bar{\psi}_3\gamma_5\gamma^\mu\psi_4) + g_{\mu\nu}(\bar{\psi}_1\sigma^{\mu\nu}\psi_2)(\bar{\psi}_3\sigma^{\mu\nu}\psi_4),\end{aligned} \tag{S48}$$

where g_s, g_5, g_μ, $g_{5\mu}$ and $g_{\mu\nu}$ are coupling constants. Eq. (S48) is a part of Hamiltonian and should be scalar under Lorentz transformation. Hence any term of form $(\bar{\psi}_1\Gamma\psi_2)(\bar{\psi}_3\Gamma\psi_4)$ which Γ is one of the sixteen basis γ matrices, is a scalar. The Fierz transformation shows that these terms are not linearly independent and when the sequence of two of the spinors exchanged, there is a relation between products of bilinears.

For showing that, we note that the set of 16 matrices $\{\mathbb{1}, \gamma^\mu, \sigma^{\mu\nu}, \gamma^\mu\gamma^5, \gamma^5\}$ forms a complete basis that any 4 by 4 matrix can be written as a linear combination of these 16 matrices:

$$\gamma = \sum_{a=1}^{16} c_a\gamma_a, \tag{S49}$$

where $c_a = \frac{1}{16}Tr[\gamma_a\gamma_a]Tr[\gamma\gamma_a]$. Replacing c_a in (S49), we have:

$$\gamma = \frac{1}{16}\sum_{a=1}^{16} Tr[\gamma_a\gamma_a]Tr[\gamma\gamma_a]\gamma_a, \tag{S50}$$

or if we write the matrix elements of γ,

$$\gamma^i_k = \frac{1}{16}\sum_{a=1}^{16} Tr[\gamma_a\gamma_a]\gamma^l_m\gamma^m_{a\ell}\gamma^i_{ak}, \tag{S51}$$

which we use Einstein summation notation means m is a dummy index varies from 1 to 16. Appearing γ^i_k on the left and γ^ℓ_m on the right side, gives the result that,

$$\frac{1}{16}\sum_{a=1}^{16} Tr[\gamma_a\gamma_a]\gamma^m_{a\ell}\gamma^i_{ak} = \delta^m_k\delta^i_\ell. \tag{S52}$$

Now if we multiple $\Gamma^{m'}_m\Gamma'^{\ \ell}_{\ell'}$ to two sides of (S52), where Γ and Γ' can be any of 16 gamma matrices, we have,

$$\frac{1}{16}\sum_{a=1}^{16} Tr[\gamma_a\gamma_a](\Gamma\gamma_a\Gamma')^m_{\ell'}\gamma^i_{ak} = \Gamma^{m}_k{}'\Gamma'^{\ i}_{\ell'}. \tag{S53}$$

Multiplying $\bar{\psi}^k_1$, $\psi_{2m'}$, $\bar{\psi}_3^{\ell'}$ and ψ_{4i},

$$\frac{1}{16}\sum_{a=1}^{16} Tr[\gamma_a\gamma_a]\bar{\psi}^{\ell'}_3(\Gamma\gamma_a\Gamma')^m_{\ell'}\psi_{2m'}\bar{\psi}^k_1\gamma^i_{ak}\psi_{4i} = \bar{\psi}^k_1\Gamma^{m'}_k\psi_{2m'}\bar{\psi}^{\ell'}_3\Gamma'^{\ i}_{\ell'}\psi_{4i}, \tag{S54}$$

which means, ψ_{2m} and ψ_{4i}, when come from the left side to the right side of the equation, change their partners. This result was obtained only according to the indices. Now if we substitute any of the Dirac matrices for Γ and Γ', the relation between the terms of (S48) are obtained which can be expressed as [3]:

$$(\bar{\psi}_1\Gamma_i\psi_2)(\bar{\psi}_3\Gamma^i\psi_4) = \sum_k c_{ik}(\bar{\psi}_1\Gamma_k\psi_2)(\bar{\psi}_3\Gamma^k\psi_4). \tag{S55}$$

c_iks are the elements of Fierz table, which show the quadrants in Eq (S48) are linearly dependent(see Fig. ??).

For the first row of this table, we need to choose Γ and Γ' in Eq. (S54) as unit matrix $\mathbb{1}$, and γ_a is different members of 16 basis matrices. By this choice, we have:

$$\frac{1}{16}\sum_{a=1}^{16} Tr[\gamma_a\gamma_a]\bar{\psi}^{\ell'}_3(\gamma_a)^{m'}_{\ell'}\psi_{2m'}\bar{\psi}^k_1\gamma^i_{ak}\psi_{4i} = \bar{\psi}^k_1\delta^{m'}_k\psi_{2m'}\bar{\psi}^{\ell'}_3\delta^i_{\ell'}\psi_{4i}. \tag{S56}$$

Product	$(\bar{\psi}_1\psi_4)(\bar{\psi}_3\psi_2)$	$(\bar{\psi}_1\gamma_\mu\psi_4)(\bar{\psi}_3\gamma^\mu\psi_2)$	$(\bar{\psi}_1\sigma_{\mu\nu}\psi_4)(\bar{\psi}_3\sigma^{\mu\nu}\psi_2)$	$(\bar{\psi}_1\gamma_\mu\gamma_5\psi_4)(\bar{\psi}_3\gamma^\mu\gamma^5\psi_2)$	$(\bar{\psi}_1\gamma_5\psi_4)(\bar{\psi}_3\gamma^5\psi_2)$
$(\bar{\psi}_1\psi_2)(\bar{\psi}_3\psi_4)$	1/4	1/4	-1/4	-1/4	1/4
$(\bar{\psi}_1\gamma_\mu\psi_2)(\bar{\psi}_3\gamma^\mu\psi_4)$	1	-1/2	0	-1/2	-1
$(\bar{\psi}_1\sigma_{\mu\nu}\psi_2)(\bar{\psi}_3\sigma^{\mu\nu}\psi_4)$	-3/2	0	-1/2	0	-3/2
$(\bar{\psi}_1\gamma_\mu\gamma_5\psi_2)(\bar{\psi}_3\gamma^\mu\gamma^5\psi_4)$	-1	-1/2	0	-1/2	1
$(\bar{\psi}_1\gamma_5\psi_2)(\bar{\psi}_3\gamma^5\psi_4)$	1/4	-1/4	-1/4	1/4	1/4

TABLE S1. Fierz table: The identities for decomposition of a product of two Dirac bilinears of the same type to other products of bilinears when the sequence of the spinors are exchanged.

Hence,

$$\frac{1}{16}\sum_{a=1}^{16} Tr[\gamma_a\gamma_a]\bar{\psi}_3^i(\gamma_a)_i^k\psi_{2k}\bar{\psi}_1^k(\gamma_a)_k^i\psi_{4i} = \bar{\psi}_1^k\psi_{2k}\bar{\psi}_3^i\psi_{4i}. \tag{S57}$$

$Tr[\gamma_a\gamma_a]$ is 4 for $\gamma_a = \mathbb{1}, \gamma^0, \gamma^5, \gamma^1\gamma^5, \gamma^2\gamma^5, \gamma^3\gamma^5, \sigma^{01}, \sigma^{02}, \sigma^{03}$ and is -4 for $\gamma_a = \gamma^1, \gamma^2, \gamma^3, \gamma^0\gamma^5, \sigma^{12}, \sigma^{23}, \sigma^{31}$, so the left part of Eq. (S56) reduces to

$$\begin{aligned}(\bar{\psi}_1\psi_2)(\bar{\psi}_3\psi_4) \;=\; & 1/4(\bar{\psi}_1\psi_4)(\bar{\psi}_3\psi_2) + 1/4(\bar{\psi}_1\gamma_\mu\psi_4)(\bar{\psi}_3\gamma^\mu\psi_2) - 1/4(\bar{\psi}_1\sigma_{\mu\nu}\psi_4)(\bar{\psi}_3\sigma^{\mu\nu}\psi_2) \\ & -1/4(\bar{\psi}_1\gamma_\mu\gamma_5\psi_4)(\bar{\psi}_3\gamma^\mu\gamma^5\psi_2) + 1/4(\bar{\psi}_1\gamma_5\psi_4)(\bar{\psi}_3\gamma^5\psi_2)\end{aligned} \tag{S58}$$

The only thing which we should pay attention is that in Eq.S55 the γ matrices are appeared as Γ_i and Γ^i means one of them with upper index and the later with lower index. But in Eqs. S56 an S57 the indices are both upper indices. this difference leads to an extra negative sign in tensor and pseudo-vector terms. For vector terms, $Tr[\gamma^j\gamma^j]$ with $j = 1, 2, 3$ is -4 but $\gamma^j = -\eta_{jj}\gamma_j$ where $\eta_{jj} = -1$. For 0-component of vector terms, the trace part is $+1$ and η_{00} is also $+1$ so similar to other components of vector terms, the coefficient of this term is $+1/4$. For $\gamma^\mu\gamma^5$ with $\mu = 1, 2$ and 3, the trace part is positive and $\gamma^\mu\gamma^5 = -\gamma_\mu\gamma_5$ but if $\mu = 0$, lowering the indices does not change the sign of the term but $Tr[(\gamma^0\gamma^5)^2]$ is -1. So the coefficients of all pseudo-vectors is $-1/4$. The same argument works for tensor terms, keeping in mind that $\sigma^{\mu\nu}$ is $-\sigma_{\mu\nu}$ for $\mu = 0$ and $\nu = 1, 2, 3$ and is $+\sigma_{\mu\nu}$ for other Tensor indices.

Now for the second row of Fierz table, we have to go through all the previous steps but with choosing Γ and Γ' in Eq. (S54) as γ^μ and γ_μ. Therefore, in right side, we have $\gamma^\mu\gamma_a\gamma_\mu$ which have different values depending on different γ_a. For $\gamma_a = \mathbb{1}$, it would be $\gamma_\mu\gamma_m u$ which is 4 and so gets the value 1 for the coefficient of scalar term in expanding $(\bar{\psi}_1\gamma^\mu\psi_2)(\bar{\psi}_3\gamma_\mu\psi_4)$ in terms of different quadrants. For the vector term, $\gamma_a = \gamma^\mu$ and according to Fierz identities, $\gamma^\mu\gamma_\mu\gamma_\mu = -2\gamma_\mu$. This leads to coefficient $-1/2$ in V-column of the second row. For T-column, we set $\gamma_a = \sigma^{\mu\nu}$ and $\gamma^\mu\sigma_{\mu\nu}\gamma_\mu$ is 0. The details of this calculation are very straightforward and only include commuting of γ_μ and $\sigma^{\mu\nu}$ according to their upper and lower indices, so that γ^μ is placed near γ_μ. For A-column, its enough to know $\gamma^\mu\gamma^5\gamma^\mu\gamma_\mu = 2\gamma^5\gamma^\mu$ and for the last column $\gamma^\mu\gamma^5\gamma_\mu = -4\gamma^5$.

Other rows of the table are also obtained with the same trick and only by commuting γ matrices.

---

[1] A. Zee, *Quantum Field Theory in a Nutshell* (Princeton University Press, Princeton, 2010).
[2] T. Kita, *Statistical Mechanics of Superconductivity* (Springer Japan, 2015).
[3] L. Okun, *Leptons and Quarks*, North-Holland Personal Library (Elsevier Science, 2013).
